# Prevention of mitochondrial impairment by inhibition of protein phosphatase 1 activity in amyotrophic lateral sclerosis

**DOI:** 10.1038/s41419-020-03102-8

**Published:** 2020-10-21

**Authors:** So Yoen Choi, Ju-Hyun Lee, Ah-Young Chung, Youhwa Jo, Joo-ho Shin, Hae-Chul Park, Hyun Kim, Rodrigo Lopez-Gonzalez, Jae Ryun Ryu, Woong Sun

**Affiliations:** 1 Department of Anatomy, Korea University College of Medicine, Brain Korea 21 plus, Seoul, 02841 Republic of Korea; 2 Department of Neurology, University of Massachusetts Medical school, Worcester, MA USA; 3 Graduate School of Medicine, Korea University, Ansan, Gyeonggido Republic of Korea; 4 Division of Pharmacology, Department of Molecular Cell Biology, Samsung Biomedical Research Institute, Sungkyunkwan University School of Medicine, Suwon, Gyeonggi-do 440-746 Republic of Korea

**Keywords:** Mitochondria, Amyotrophic lateral sclerosis, Amyotrophic lateral sclerosis

## Abstract

Amyotrophic lateral sclerosis (ALS) is a fatal neurodegenerative disease caused by progressive loss of motor neurons (MNs) and subsequent muscle weakness. These pathological features are associated with numerous cellular changes, including alteration in mitochondrial morphology and function. However, the molecular mechanisms associating mitochondrial structure with ALS pathology are poorly understood. In this study, we found that Dynamin-related protein 1 (Drp1) was dephosphorylated in several ALS models, including those with SOD1 and TDP-43 mutations, and the dephosphorylation was mediated by the pathological induction of protein phosphatase 1 (PP1) activity in these models. Suppression of the PP1-Drp1 cascade effectively prevented ALS-related symptoms, including mitochondrial fragmentation, mitochondrial complex I impairment, axonal degeneration, and cell death, in primary neuronal culture models, iPSC-derived human MNs, and zebrafish models in vivo. These results suggest that modulation of PP1-Drp1 activity may be a therapeutic target for multiple pathological features of ALS.

## Introduction

Amyotrophic lateral sclerosis (ALS) is a neurodegenerative disorder characterized by progressive and selective loss of motor neurons (MNs)^1,2^. The clinical symptoms of ALS include muscle weakness, dysphagia, and speech problems, and the disorder eventually leads to death from dyspnea within three to 5 years of diagnosis^3,4^. Most ALS cases are sporadic, and roughly 10% of them are familial forms caused by various genetic mutations in specific genes, including superoxide dismutase 1 (SOD1), transactive response DNA-binding protein-43 (TDP-43), and chromosome 9 open reading frame 72 (C9orf72)^5,6^. Mutations in these genes are also found in the sporadic form of ALS^1^. For example, hexanucleotide repeat expansion of C9orf72 was identified in a large proportion of both familial and sporadic forms of ALS patients, suggesting that somatic or de novo mutations in causative genes may also be responsible for sporadic ALS^7,8^. Although the causes of ALS may differ, most ALS cases exhibit common pathological features with biochemical/cellular hallmarks such as protein aggregations, abnormal Ca^2+^ buffering^9^, glutamate excitotoxicity^10^, glial activation^11^, disruption of RNA metabolism^12^, and mitochondrial dysfunction^13,14^. Therefore, it has been postulated that multiple cellular changes are involved in the progression of ALS symptoms, making it difficult to develop effective treatments. Although riluzole and edaravone are the drugs for ALS currently approved by FDA, their efficacies are modest in clinical trials, and their pharmacological targets appear to be different^15–17^. It is, therefore, of great importance to develop novel treatments targeting several of the common pathological features responsible for the complex nature of ALS pathology.

Mitochondrial dysfunction is commonly found in both familial and sporadic ALS cases^18–20^, suggesting that mitochondria may serve as an important hub for ALS pathophysiology. For example, it has been reported that mitochondria were morphologically fragmented and functionally deteriorated from the pre-symptomatic period in patients and mouse models of ALS (SOD1 G93A)^21,22^. C9ORF72-associated poly(GR) compromises mitochondrial function and induces mitochondrial fragmentation, leading to DNA damage and an increase in cell death^23,24^. In sporadic ALS cases, mitochondrial morphological abnormalities have been detected in the anterior horn of the spinal cord^19^. Mitochondria are essential for cellular ATP production, and MNs require large amounts of energy from the mitochondria in order to function. Thus, it has been suggested that MNs are one of the most vulnerable types of cells to mitochondrial dysfunction^25–27^. On the other hand, the morphological changes in the mitochondria in ALS pathology may hint at additional links between mitochondria and MN function. The morphological dynamics of mitochondria are homeostatically controlled by the balance between continuous fission and fusion, and this is necessary for the maintenance of mitochondrial quality^28,29^. Mitochondrial dynamics are controlled by large GTPase dynamin-related proteins, including Dynamin-related protein 1 (Drp1), Mitofusin1/2 (MFN1/2), and Optic atrophy 1 (Opa1)^30^. Recently, it was reported that the expression of Drp1 was induced in the spinal cord of SOD1 G93A model mice^31^, and the suppression of Drp1 inhibited mutant SOD1-induced mitochondrial fragmentation and cell death in vitro^2^, suggesting that alteration of Drp1 activity may mediate at least some aspects of MN degeneration in ALS. However, the precise molecular mechanisms underlying the Drp1 function and ALS pathology are yet to be described.

In an attempt to identify novel therapeutic targets for ALS, we examined whether Drp1-dependent mitochondrial dynamics are an effective target for ALS therapy and explored the upstream mechanism modulating Drp1 activity in ALS models. To this end, we identified that Drp1 is pathologically dephosphorylated in SOD1 G93A mice, and the blockade of Drp1 activity prevents cell death induced by the expression of mutant SOD1 or TDP-43. Furthermore, we identified PP1 as a phosphatase responsible for the dephosphorylation of Drp1 and found that the inhibition of PP1 activity suppresses neurotoxicity in several ALS models. Based on our observations, we propose that pathological induction of PP1 plays a key role in the progression of cellular events associated with ALS, providing novel therapeutic targets for ALS.

## Materials and methods

### Animal model and treatment

SOD1 G93A transgenic mice were maintained as a hemizygous line by mating with wild type C57BL/6J mice. To distinguish between SOD1 G93A and WT mice, pups were genotyped with DNA obtained from their toes, using the PCR method. To create the ALS zebrafish models, embryos from *Tg*(*olig2-egfp*) zebrafish, whose MN axons exhibit EGFP^32,33^, were microinjected with 1.76 ng RNA (SOD1 G93A and TDP-43 Q331K), synthesized by T7 RNA polymerase. Embryos were treated with Mdivi-1 (2.5 nM, Sigma, Cat. 338967-87-6), I-2 (200 nM, Millipore, Cat. 14-162), or Okadaic acid (100 nM, Sigma, Cat. O8010) 24 h after microinjection, and then once again the following day. The animal models were randomly assigned to experimental groups.

### Primary cortical neuron culture

The Cerebral cortices were dissected from WT rat or mouse embryos (E16~17) and chopped using a micro scissor. The tissues were incubated in 0.05% Trypsin-EDTA at 37 °C for 10 min and then centrifuged at 1000 rpm for 1 min. The dissociated cells were washed with HBSS and re-suspended in complete medium, containing Neurobasal^TM^ medium (Invitrogen, Cat. 21103) supplemented with B27 (GIBCO BRL, Cat. 17504044), L-glutamic acid (GIBCO BRL), L-glutamine (GIBCO BRL), and penicillin–streptomycin (GIBCO BRL). The cells were plated onto poly-D lysine pre-coated coverslips (2 × 10^5^ cells/well) and cultured in complete medium. After 3 days, the complete medium was replaced with incomplete medium, containing Neurobasal^TM^ medium supplemented with B27, L-glutamine, and penicillin–streptomycin. On 4 days in vitro, 50 μM Mdivi-1 or I-2 (NEB, Cat. P0755L) were treated for 24 or 72 h. In some cases, shDrp1 (5'-GAA GAG TGT AAC TGA TTC A-3’) or shFis1 (5'-CAG CGG GAT TAT GTC TTC T-3’) sequences were inserted into the BglII/HindIII site of the p-Super GFP vector. shPP1α (5'-GCA GCT GAC AGA GAA CGA GAT-3’), shPP1β (5'-GCT AAA CGA CAG TTG GTA ACC-3’) or shPP1γ (5'-GGG TAT GAT CAC AAA GCA AGC-3’) sequences were inserted into the PacI site of the FUGW-GFP vector, and the resultant vectors were cotransfected with ALS-related mutants (SOD1 G93A, TDP-43 Q331K). Three days after transfection, the pyknotic and activated caspase-3 labeled cells were counted.

### MN differentiation and pharmacological treatments

Differentiation of iPSCs into MNs was performed as described by Lopez-Gonzalez et al.^21^. Briefly, iPSCs were plated and expanded in an mTSER1 medium (Stem Cell Technologies Cat. 5875) in Matrigel-coated wells (Corning Cat. 354230). Twenty-four hours after plating, the culture medium was replaced with neuroepithelial progenitor (NEP) medium, containing DMEM/F12 (Invitrogen Cat. 12660-012):neurobasal medium (Invitrogen Cat. 21103049) at 1:1, 0.5 × N2 (Thermo-Fisher Cat. 17502-048), 0.5 × B27 (Thermo-Fisher Cat. 17504-044), 0.1 mM ascorbic acid (Sigma Cat. A4403), 1 × Glutamax (Invitrogen35050061), 3 μM CHIR99021 (Stem Cell Technologies Cat. 72054), 2 μM DMH1 (Stem Cell Technologies Cat. 73634), and 2 μM SB431542 (Stemgent Cat. 040010-10). After 6 days, NEPs were dissociated with accutase, split 1:6 into Matrigel-coated wells, and cultured for 6 days in motor neuron progenitor induction medium (NEP) with 0.1 μM retinoic acid (Sigma Cat. R2625) and 0.5 μM purmorphamine (Calbiochem Cat. 540220). Motor neuron progenitors were dissociated with accutase to generate neurosphere suspension cultures. After a further 6 days, the cultures were dissociated into single cells, plated on laminin-coated plates/coverslips (TTE laboratories Cat. WN354087) in a motor neuron differentiation medium containing 0.5 μM retinoic acid, 0.1 μM purmorphamine, and 0.1 μM Compound E (Stem Cell Technologies Cat. 73954) for 2 weeks and then in the same medium without Compound E for up to 3 weeks. iPSC-derived motor neurons from controls lines 35L11, 37L20, and 24L2, and TDP-43 mutant lines 292 (M337V), 290 (Q343R), and 36L10 (A90V) were treated from 1 week after plating with I-2 for an additional 2 weeks, with the medium changed twice a week. After that, the cells were fixed for imaging analysis.

### Western blot

The lumbar region of the spinal cord was collected from WT and SOD1 G93A mice and sonicated in a lysis buffer (50 mM Tris-Cl pH 7.4, 150 mM NaCl, 10% Glycerol, 1% Triton-X100 and 1 mM EDTA). After protein quantification using the BCA method, 30 µg of protein was loaded per well and separated by SDS-PAGE. The loaded protein was transferred to a PVDF membrane, and this membrane was incubated in a blocking solution (3% BSA/1 ×TBST) for 1 h. The membrane was incubated with primary antibody diluted 1:1000 with blocking solution overnight at 4 °C, then washed three times in 1xTBST and incubated with secondary antibody diluted 1:5000 with 5% Skim Milk/1xTBST. The antibodies used were anti-Drp1 (BD, Cat. 611113), anti-phospho Drp1 S616 (Cell signaling, Cat. 3455), anti-Fis1 (Abcam, Cat. Ab96764), anti-PP1α (Santa Cruz, Cat. sc-443), anti-phospho-PP1α (Cell Signaling, Cat. #2581), PP1β (Santa Cruz, Cat. sc-373782), PP1γ (Santa Cruz, Cat. sc-6109), and anti β-actin (Sigma, Cat. A5441). The secondary antibodies used were anti-mouse and anti-rabbit IgG conjugated to horseradish peroxidase. The membrane was washed three times in 1xTBST, and the signals expressed by the protein were visualized using an ECL kit (Thermo, Cat. 32106).

### Immunohistochemistry

SOD1 G93A mice of each pathological stage were perfused in 4% paraformaldehyde, and the lumbar regions of the spinal cord were dissected. The tissues were post-fixed overnight at 4 °C. The fixed tissues were soaked in 30%sucrose/1× PBS for cryo-protection. The lumbar regions of the spinal cord were cut serially into sections 40 μm thick on a cryostat, and these were transferred into 50% glycerol in PBS. The sections were blocked in 1xPBS containing 3% BSA and 0.2% Triton-X100 for 30 min at room temperature. After blocking, tissues were incubated with primary antibody overnight at 4 °C and washed three times in 1× PBS. The primary antibodies used were phospho-DRP1 S616 (Cell Signaling, Cat. 3455), DRP1 (BD, Cat. 611113), and ChAT (Millipore, Cat. AB144P). The tissues were incubated with fluorescence-labeled secondary antibody and Hoechst33342 (10 μg/ml; Sigma, St. Louis, MO) for 30 min at room temperature and washed three times in 1× PBS. They were then mounted on slides and observed using a fluorescence or confocal microscope (Zeiss, Goettingen, Germany).

### PP1 activity assay

PP1 activity was measured by the ProFluor Ser/Thr PPase assay kit (Promega, Cat. V1260). This assay is based on the dephosphorylation of a phosphorylated bisamide rhodamine 110 peptide substrate. In order to carry out a PP1 activity assay of the lumbar spinal cord, the tissue was sonicated in a lysis buffer (5 M NaCl, 1 M Tris-Cl, pH 7.5, 0.5 M EDTA, 0.5 M EGTA, 0.2 M Na_3_VO_4_, 0.1 M PMSF) and centrifuged at 10,000 rpm for 10 min. Following centrifugation, the supernatant was divided into two tubes and treated with either 0.2 nM (for PP2A inhibition) or 1 μM okadaic acid (for PP1 inhibition). The level of PP1 activity of each sample was measured based on the absorbance at 488 nm of nonphosphorylated S/T PPase R110 substrate and then normalized by protein levels (BCA assay). Relative PP1 activity was calculated as the value of phosphatase activity level (treated with 0.2 nM okadaic acid) minus the value of background phosphatase activity (treated with 1 μM okadaic acid)^34^.

### Assay of NADH dehydrogenase and complex I activity of mitochondria

In order to carry out a histological assay of NADH dehydrogenase, the lumbar spinal cord was snap-frozen in pre-chilled isopentane and sectioned using a cryostat. Phosphate buffer (0.343 g NaH_2_PO_4_ in 100 ml H_2_O) and NBT (250 mg nitro blue tetrazolium in 100 ml H_2_O) were prepared. The sections were incubated in a solution containing 5 mg NADH, 1 ml saline, 1 ml phosphate buffer, and 4 ml NBT with 2 ml distilled water. After incubation for 30 min at room temperature in humidity chamer, the sections were rinsed in distilled water and observed using a microscope. The mitochondrial complex I activity of the lumbar spinal cord and cultured neurons was measured using a microplate assay kit (Abcam, Cat. Ab109721) according to the manufacturer’s instruction^35^.

### Measurement of mitochondrial membrane potential (∆Ψ_m_) and velocity

Cultured cortical neurons were incubated with 500 nM TMRM (tetramethylrhodamine, methyl ester; Invitrogen, Cat. T668) for 30 min at 37 °C. The signal of TMRM was imaged using a Carl Zeiss Observer Z1 with x630 magnification, and its intensity was measured using ImageJ.

### Statistical analysis

All samples or animals were assigned randomly, and the experiment was performed in a blinded fashion. The data were tested for differences between multiple groups using one-way ANOVA followed by Scheffe’s or Tukey’s multiple comparisons tests for post hoc comparisons. Differences between the means of two groups were tested using the two-sided Student’s *t*-test.

### Ethical statements

All animal maintenance and experimental procedures were approved by members of the Laboratory Animal Research Center at Korea University College of Medicine. The use of hiPSCs was approved by the Institutional Review Board and Ethics Committee at the University of California, San Francisco (UCSF).

## Results

### Changes in mitochondrial fission proteins in ALS models

**Fig. 1:**
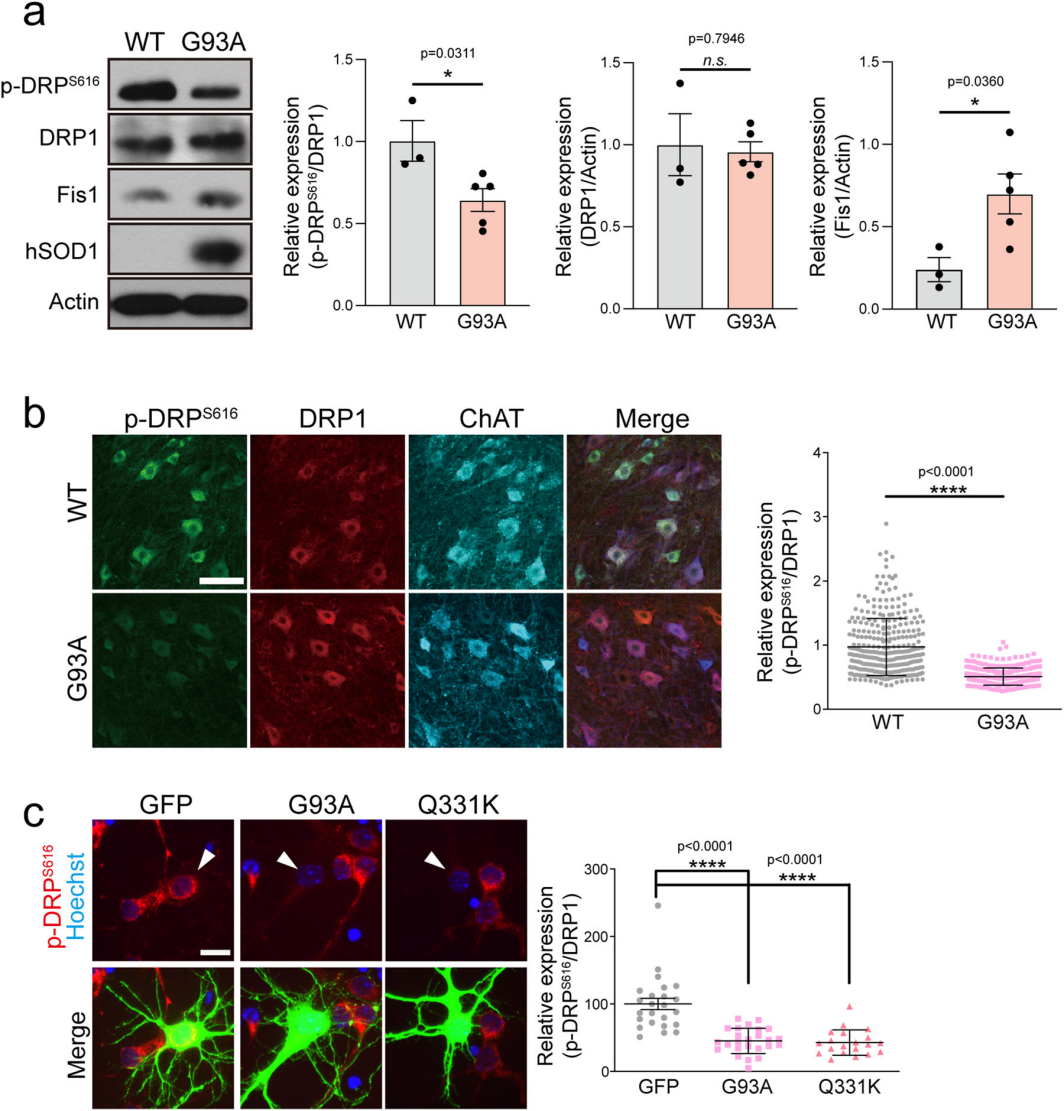
Reduction of Drp1 S616 phosphorylation in the SOD1 G93A model. **a** p-Drp1 S616 and Fis1 protein expression levels in the lumbar spinal cord of SOD1 G93A mice, and quantification of the relative expression of p-Drp1 S616 and Fis1, compared to WT. WT:p-DRP^S616^ = 1.00 ± 0.12 (*n* = 3 mice), G93A: p-DRP^S616^ = 0.64 ± 0.07 (*n* = 5 mice), WT:DRP1 = 1.00 ± 0.12 (*n* = 3 mice), G93A: DRP1 = 0.96 ± 0.06 (*n* = 5 mice), WT:Fis1 = 0.24 ± 0.07 (*n* = 3 mice), G93A: Fis1 = 0.70 ± 0.12 (*n* = 5 mice). Values are mean ± S.E.M. F(1,6) = 2.80, *P = 0.0311 for p-DRP^S616^; F(1,6) = 0.2721, *P* = 0.7946 for DRP1; F(1,6) = 2.691, *P = 0.0.0360 for Fis1 graph by two-sided Student’s *t*-test. *n.s.:* not significant. **b** p-Drp1 S616 immunostaining in the spinal MN of G93A and WT mice. The graph shows the ratio of intensity between p-Drp1 (green) and Drp1 (red) in ChAT-positive (blue; MN marker) neurons. WT = 1.56 ± 0.04 (*n* = 316 ChAT+ neurons), G93A = 0.81 ± 0.01 (*n* = 346 ChAT+ neurons. Values are mean **±** S.E.M. F(1,660) = 18.4, ****P < 0.0001 by two-sided Student’s *t*-test. Scale bar, 100 μm. **c** Expression of p-Drp1 S616 in GFP-positive cortical neurons, following G93A or Q331K overexpression. The graph shows the quantification of p-Drp1 S616 intensity. Arrowhead indicates a GFP-positive neuron. GFP = 100.00 ± 8.40 (*n* = 24 GFP+ neurons), G93A = 45.30 ± 3.73 (*n* = 25 GFP+ neurons), Q331K = 42.95 ± 4.2 (*n* = 20 GFP+ neurons). Values are mean ± S.E.M. F(2,66) = 29.75, ****P < 0.0001 by one-way ANOVA with Tukey’s post hoc analysis for multiple comparisons. Scale bar, 20 μm.

We first explored whether molecules responsible for the morphological dynamics of mitochondria are altered in SOD1 G93A (G93A) ALS model mice. Although the expression level of Drp1 protein remained unaltered, the phosphorylation level of Drp1 S616 was significantly reduced at the onset period (when the mice were 100 days old) (Fig. 1a). The expression level of Fis1, a receptor for Drp1, also markedly increased during this period (Fig. 1a). Immunohistochemical labeling of the lumbar spinal cord with a phosphorylation-specific antibody against Drp1 S616 demonstrated that the reduction of Drp1 S616 phosphorylation mainly occurred in the MNs of G93A mice (Fig. 1b). Next, we overexpressed G93A or TDP-43 Q331K (Q331K) associated with ALS in primary neurons and found that these pathogenic mutants caused dephosphorylation of Drp1 (Fig. 1c). Collectively, these results suggest that pathogenic stimuli associated with ALS cause dephosphorylation of Drp1.

### Prevention of Drp1 signaling reduces ALS-related neurodegeneration

**Fig. 2:**
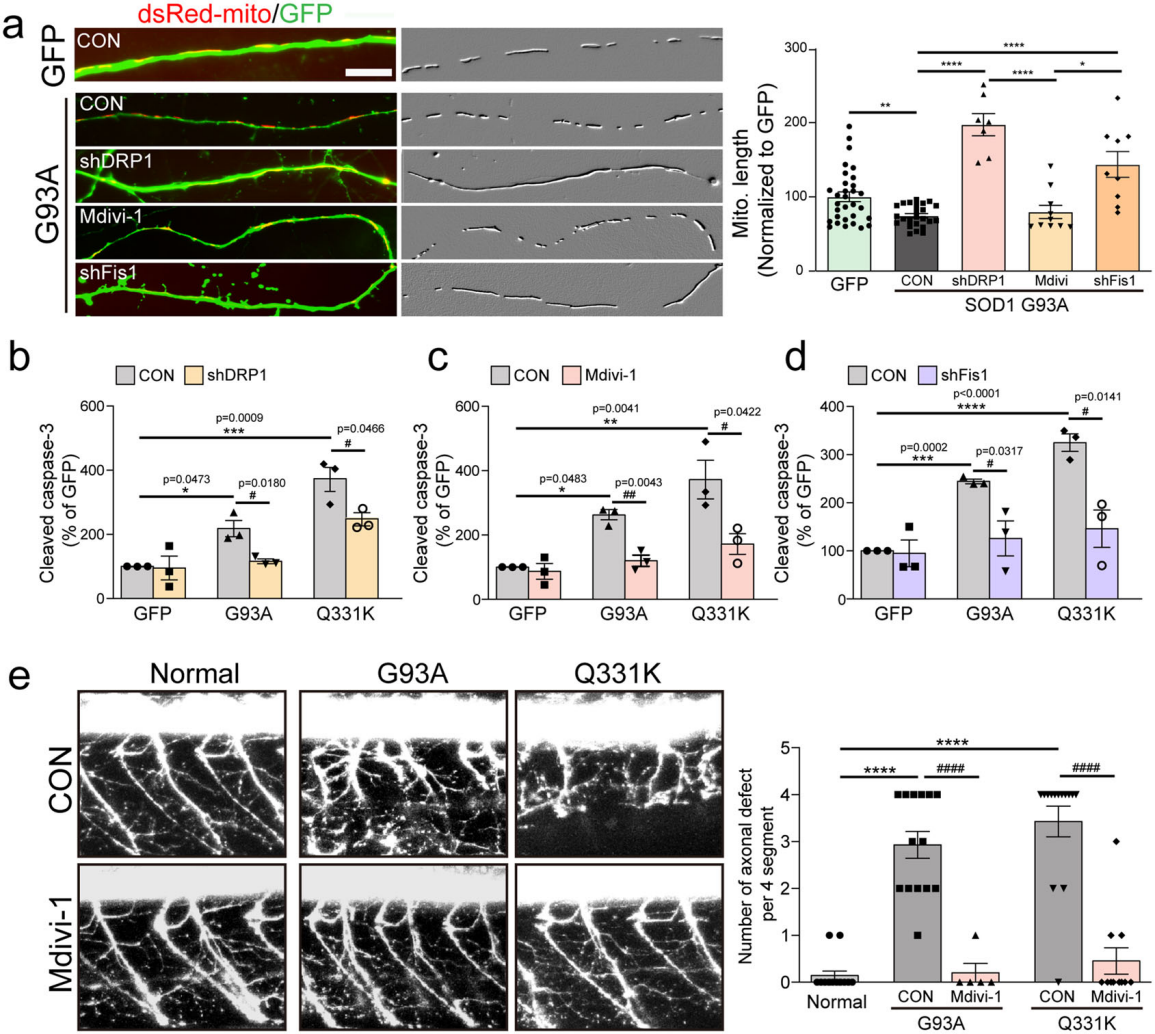
Effects of the blockade of Drp1 activity on ALS mutant-induced pathological features. Analysis of mitochondrial length ((**a**) *N* = 10 per group) and active caspase-3-positive neurons (**b**–**d**) after 3 days following shRNA co-transfection or 25 μM Mdivi-1 treatment with G93A or Q331K clone. Mitochondrial images have been embossed. GFP = 100.1 ± 6.54 (*n* = 31 GFP+ neurons), SOD G93A:CON = 74.55 ± 6.54 (*n* = 24 GFP+ neurons), SOD G93A:shDRP1 = 197.3 ± 14.96 (*n* = 7 GFP+ neurons), SOD G93A:Mdivi = 105.5 ± 8.79 (*n* = 10 GFP+ neurons), SOD G93A:shFis1 = 143.7 ± 17.38 (*n* = 9 GFP+ neurons). Values are mean **±** S.E.M. F(1,53) = 3.239, **P = 0.0021 for GFP versus SOD G93A:CON by two-sided Student’s *t*-test. F(3,46) = 33.54, *P = 0.0440, ****P < 0.0001 by one-way ANOVA with Tukey’s post hoc analysis for multiple comparisons in (**a**). GFP:CON = 100 ± 0.00 (*n* = 3 independent primary cultures), GFP:shDRP1 = 95.25 ± 36.86 (*n* = 3 independent primary cultures), G93A:CON = 218 ± 25.18 (*n* = 3 independent primary cultures), G93A:shDRP1 = 116.4 ± 7.52 (*n* = 3 independent primary cultures), Q331K:CON = 370 ± 38.99 (*n* = 3 independent primary cultures), Q331K:shDRP1 = 247.6 ± 18.38 (*n* = 3 independent primary cultures). Values are mean ± S.E.M. F(2,6) = 25.58, *P = 0.0473, ***P = 0.009 by one-way ANOVA with Tukey’s post hoc analysis for multiple comparisons. F(1,4) = 0.1289, *P* = 0.9037 for GFP:CON versus GFP:shDRP1; F(1,4) = 3.869, ^#^*P* = 0.0180 for G93A:CON versus G93A:shDRP1; F(1,4) = 2.846, ^#^*P* = 0.0466 for Q331K:CON versus Q331K:shDRP1 by two-sided Student’s *t*-test in (**b**). GFP:CON = 99.98 ± 0.03 (*n* = three independent primary cultures), GFP:Mdivi-1 = 86.85 ± 24.57 (*n* = 3 independent primary cultures), G93A:CON = 257 ± 15.85 (*n* = 3 independent primary cultures), G93A: Mdivi-1 = 119.9 ± 17.42 (*n* = 3 independent primary cultures), Q331K:CON = 372 ± 60.06 (*n* = 3 independent primary cultures), Q331K: Mdivi-1 = 171.9 ± 32.03 (*n* = 3 independent primary cultures). Values are mean ± S.E.M. F(2,6)=14.53, *P = 0.0483, **P = 0.0041 by one-way ANOVA with Tukey’s post hoc analysis for multiple comparisons. F(1,4) = 0.5345, *P* = 0.6213 for GFP:CON versus GFP:shDRP1; F(1,4) = 5.819, ^##^*P* = 0.0043 for G93A:CON versus G93A:shDRP1; F(1,4) = 2.922, ^#^*P* =0 .0422 for Q331K:CON versus Q331K:shDRP1 by two-sided Student’s *t*-test in (**c**). GFP:CON = 100 ± 0.03 (*n* = 3 independent primary cultures), GFP:shFis1 = 94.73 ± 27.67 (*n* = 3 independent primary cultures), G93A:CON = 244 ± 4.88 (*n* = 3 independent primary cultures), G93A:shFis1 = 125.8 ± 36.28 (*n* = 3 independent primary cultures), Q331K:CON = 324.9 ± 18.34 (*n* = 3 independent primary cultures), Q331K: shFis1 = 146 ± 38.89 (*n* = 3 independent primary cultures). Values are mean ± S.E.M. F(2,6) = 108.1, ***P = 0.0002, ****P < 0.0001 by one-way ANOVA with Tukey’s post hoc analysis for multiple comparisons. F(1,4) = 0.1922, *P* = 0.8569 for GFP:CON versus GFP:shDRP1; F(1,4) = 3.24, ^#^*P* = 0.0317 for G93A:CON versus G93A:shDRP1; F(1,4) = 2.922, ^#^*P* = 0.0141 for Q331K:CON versus Q331K:shDRP1 by two-sided Student’s *t*-test in (**d**). Scale bar, 5 μm. **e** Zebrafish expressing G93A and Q331 with 2.5 μM Mdivi-1 daily treatment over 2 days, and the number of axonal defects of MNs counted for each of the four segments. Normal = 0.14 ± 0.1 (*n* = 14 zebrafish), G93A:CON = 2.93 ± 0.29 (*n* = 14 zebrafish), G93A:Mdivi-1 = 0.2 ± 0.2 (*n* = 5 zebrafish), Q331K:CON = 3.43 ± 0.33 (*n* = 14 zebrafish), Q331K:Mdivi-1 = 0.45 ± 0.28 (*n* = 11 zebrafish). Values are mean ± S.E.M. F(2,39) = 47.44, ****P < 0.0001 by one-way ANOVA with Tukey’s post hoc analysis for multiple comparisons.; F(1,17) = 5.445, ^####^*P* < 0.0001 for G93A:CON versus G93A:Mdivi-1, F(1,23) = 6.671, ^####^*P* < 0.0001 for Q331K:CON versus Q331K: Mdivi-1 by two-sided Student’s *t*-test.

It has been reported that phosphorylation of Drp1 S616 is controlled by cdk5, which prevents the oligomerization and subsequent mitochondrial localization of Drp1 in neurons^36^. Dephosphorylation of Drp1 S616 and the induction of Fis1 expression appear to promote Drp1-dependent mitochondrial fission, which is associated with neurodegeneration. In keeping with this, we found that the overexpression of mutant G93A in primary cortical neurons promoted mitochondrial fragmentation. However, suppression of Drp1 activation by transfection with shDrp1 or shFis1, and treatment with a chemical inhibitor (Mdivi-1), consistently prevented the mitochondrial fragmentation induced by ALS-related mutations, suggesting that Drp1-Fis1 signaling is necessary for ALS-related mitochondrial fragmentation (Fig. 2a and Supplementary Fig. 1a). Next, we tested whether a blockade of Drp1-Fis1 would prevent neurodegeneration. Following transfection with ALS-related mutant genes (SOD1 G93A and TDP-43 Q331K), neuronal death was enhanced, as assessed by activated caspase-3 immunoreactivity (Fig. 2b–d) and pyknotic nuclei (Supplementary Fig. 1b–d). Blocking Drp1 activation effectively prevented this cell death. Finally, we tested the effects of a blockade of Drp1 activity in vivo using an ALS zebrafish system (Fig. 2e). Degeneration of motor axons induced by transient overexpression of SOD1 G93A or TDP-43 Q331K was readily detectable in the models, but the blockade of Drp1 activity through treatment with Mdivi-1 effectively prevented this axonal degeneration. These results indicate that Drp1 activation contributes to ALS-related neurodegeneration in vivo.

### PP1 is involved in Drp1 dephosphorylation in G93A mice

**Fig. 3:**
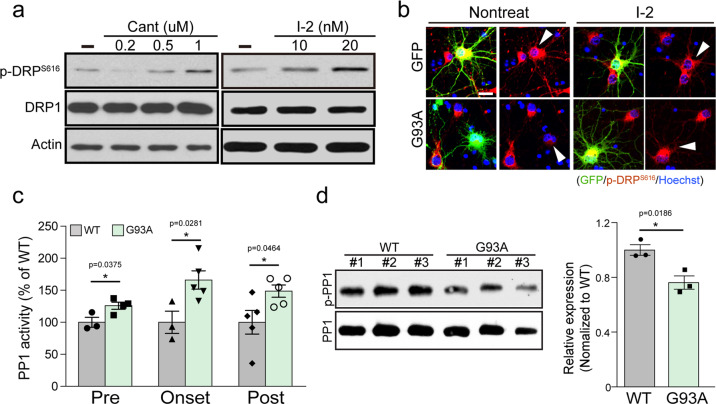
Dephosphorylation of the site S616 of Drp1 by PP1 and the increase in PP1 activity in the lumbar spinal cord of G93A mice. **a** Western blot analysis of p-Drp1 S616 and Drp1 expression level in primary cortical neuron with Cantharidin (for 1 h) or I-2 (for 1 day) treatment. **b** Representative immunostaining image of p-Drp1 S616 (red) in GFP+ primary cortical neuron with or without 80 nM I-2 treatment for 24 h. Arrowhead indicates the GFP-positive neuron. Scale bar, 20 μm. **c** PP1 activity in the lumbar spinal cord of G93A and WT mice at 60, 100, and 200 days of age. WT:Pre = 100 ± 7.79 (*n* = 3 mice), G93A:Pre = 125.9 ± 5.51 (*n* = 4 mice), WT:Onset = 100 ± 17.35 (*n* = 3 mice), G93A: Onset = 166 ± 14.32 (*n* = 5 mice), WT:Post = 100 ± 18.35 (*n* = 5 mice), G93A:Post = 148.7 ± 9.58 (*n* = 5 mice). Values are mean ± S.E.M. F(1,5) = 2.811, *P = 0.0375 for WT:Pre versus G93A:Pre; F(1,6) = 2.88, *P = 0.0281 for WT:Onset versus G93A:Onset; F(1,8) = .354, *P = 0.0464 for WT:Post versus G93A:Post by two-sided Student’s *t*-test. **d** Western blot analysis of p-PP1 and PP1 expression in the lumbar spinal cord of G93A and WT mice at the onset period. The graph shows the quantification of p-PP1 vs. PP1 intensity. WT = 1.00 ± 0.04 (*n* = 3 mice), G93A = 0.76 ± 0.05 (*n* = 3 mice). Values are mean ± S.E.M. F(1,4) = 3.832, *P = 0.0186 by two-sided Student’s *t*-test.

Because dephosphorylation of Drp1 was found to be one of the events associated with enhanced mitochondrial fission and neurodegeneration in the ALS models, we explored the upstream phosphatase(s) responsible for this dephosphorylation, under the assumption that upstream phosphatase activation may cause the Drp1-dependent mitochondrial defects found in ALS models (Fig. 3). The blockade of PP1 by either of two selective inhibitors, cantharidin or inhibitor-2 (I-2), promoted the phosphorylation of Drp1 S616 in a dose-dependent manner (Fig. 3a). Furthermore, the dephosphorylation of Drp1 was completely blocked by I-2 treatment in cortical neurons through G93A overexpression (Fig. 3b). These observations suggest that the activation of PP1 is responsible for the Drp1 dephosphorylation associated with ALS pathology. Moreover, we found that the lumbar spinal cord of G93A mice exhibited significantly higher PP1 activity in the symptomatic period than in the pre-symptomatic period, and the enhanced PP1 activity was maintained until the end stage (Fig. 3c). It has been reported that PP1 activity is regulated by its phosphorylation status, and dephosphorylation is required for its activation^37,38^. Western blot analysis showed that PP1α was activated in the pre-symptomatic period of ALS (Fig. 3d) and the phosphorylation of PP1 was decreased in the ChAT+ neurons of G93A mouse at pre- and post-symptomatic period, as well as in mSOD1 or TDP43 expressing cortical neurons (Supplementary Fig. 2). Collectively, these results demonstrated that pathologic induction of PP1 activity may cause Drp1 activation and subsequent induction of cell death in ALS models.

### PP1 inhibition rescues neurons from neurodegeneration

**Fig. 4:**
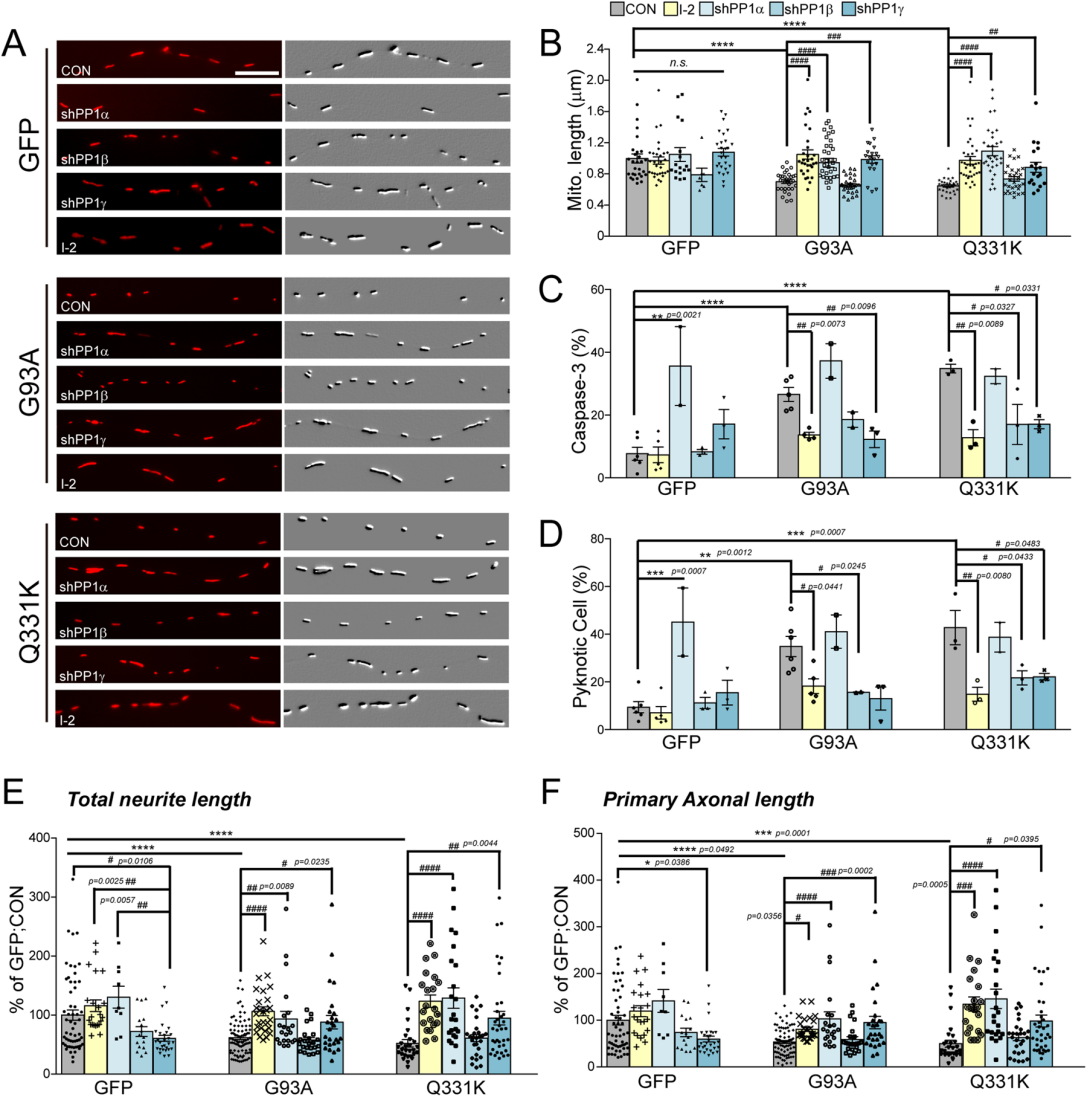
Effects of the blockade of PP1 on G93A and Q331-induced neurodegeneration. **a** The representative image of mitochondria in the neurite of GFP+ primary cortical neuron with 40 nM I-2 treatment or shRNA lenti-viral infection after transfection of dsRed-mito and mALS genes, over 72 h. The mitochondrial image has been embossed using Photoshop and measured on the GFP^+^ cortical neuron. Scale bar, 5 μm. **b** Analysis of mitochondrial length in (**a**). GFP:CON = 1.00 ± 0.06 (*n* = 29 GFP+ neurons), GFP:shPP1α = 1.1 ± 0.09 (n = 17 GFP+ neurons) GFP:shPP1β=0.79 ± 0.87 (*n* = 7 GFP+ neurons), GFP:shPP1γ = 1.08 ± 0.05 (*n* = 27 GFP+ neurons), GFP:I-2 = 0.96 ± 0.05 (*n* = 30 GFP+ neurons), G93A:CON = 0.7 ± 0.02 (*n* = 30 GFP+ neurons), G93A:shPP1α = 1.00 ± 0.4 (*n* = 33 GFP+ neurons), G93A:shPP1β = 0.66 ± 0.02 (*n* = 30 GFP+ neurons), G93A:shPP1γ = 0.99 ± 0.05 (*n* = 20 GFP+ neurons), G93A:I-2 = 1.04 ± 0.06 (*n* = 30 GFP+ neurons), Q331K:CON = 0.65 ± 0.02 (*n* = 30 GFP+ neurons), Q331K:shPP1α = 1.1 ± 0.06 (n = 31 GFP+ neurons), Q331K:shPP1β = 0.74 ± 0.03 (*n* = 30 GFP+ neurons), Q331K:shPP1γ = 0.89 ± 0.6 (*n* = 19 GFP+ neurons), Q331K:I-2 = 0.98 ± 0.05 (*n* = 35 GFP+ neurons). Values are mean ± S.E.M. F(2,86) = 26.64, ****P < 0.0001 for CON groups; F(4,105) = 1.710, *n.s*.: not significant for GFP groups; F(4,138) = 20.02, ^###^*P* = 0.0001, ^####^*P* < 0.0001 for G93A groups; F(4,138) = 20.02, ^##^*P* = 0.0089, ^####^*P* < 0.0001 for Q331K groups by one-way ANOVA with Tukey’s post hoc analysis for multiple comparisons. **c** Quantification of cleaved caspase-3-positive neurons in GFP+ neurons with 40 nM I-2 treatment or shRNA lenti-viral infection following transfection of mALS genes, over 72 h. GFP:CON = 7.67 ± 2.06 (*n* = 6 independent primary culture), GFP:shPP1α = 35.61 ± 12.54 (*n* = 2 independent primary culture), GFP:shPP1β=8.28 ± 0.75 (*n* = 3 independent primary culture), GFP:shPP1γ = 17.11 ± 4.65 (*n* = 3 independent primary culture), GFP:I-2 = 7.5 ± 2.51 (*n* = 5 independent primary culture), G93A:CON = 26.59 ± 2.31 (*n* = 5 independent primary culture), G93A:shPP1α = 37.09 ± 5.51 (*n* = 2 independent primary culture), G93A:shPP1β = 18.39 ± 2.45 (*n* = 2 independent primary culture), G93A:shPP1γ = 12.09 ± 2.63 (*n* = 3 independent primary culture), G93A:I-2 = 13.79 ± 0.84 (*n* = 4 independent primary culture), Q331K:CON = 34.83 ± 1.370 (*n* = 3 independent primary culture), Q331K:shPP1α = 32.39 ± 2.39 (*n* = 2 independent primary culture), Q331K:shPP1β = 17.03 ± 6.39 (*n* = 3 independent primary culture), Q331K:shPP1γ=17.07 ± 1.44 (*n* = 3 independent primary culture), Q331K:I-2 = 12.57 ± 2.60 (*n* = 3 independent primary culture). Values are mean ± S.E.M. F(2,11) = 41.29, ****P < 0.0001 for CON groups; F(4,14) = 7.230, ***P = 0.0007 for GFP groups; F(4,11) = 14.03, ^##^*P* = 0.0096 for G93A:CON versus G93A: shPP1γ, ^##^*P* = 0.0073 for G93A:CON versus G93A: I-2; F(4,9) = 7.887, ^#^*P* = 0.0327 for Q331K:CON versus Q331K: shPP1β, ^#^*P* = 0.0327 for Q331K:CON versus Q331K: shPP1γ, ^##^*P* = 0.0089 by one-way ANOVA followed by Tukey’s multiple comparisons test. **d** Quantification of pyknotic cell in GFP+ neurons with 40 nM I-2 treatment or shRNA lenti-viral infection following transfection of mALS genes, over 72 h. GFP:CON = 9.39 ± 2.33 (*n* = 6 independent primary culture), GFP:shPP1α = 45.01 ± 14.25 (*n* = 2 independent primary culture), GFP:shPP1β = 11.08 ± 2.31 (*n* = 3 independent primary culture), GFP:shPP1γ = 15.40 ± 5.19 (*n* = 3 independent primary culture), GFP:I-2 = 6.64 ± 2.67 (*n* = 5 independent primary culture), G93A:CON = 34.85 ± 4.25 (*n* = 6 independent primary culture), G93A:shPP1α=41.18 ± 6.97 (*n* = 2 independent primary culture), G93A:shPP1β = 15.78 ± 0.16 (n = 2 independent primary culture), G93A:shPP1γ = 13.13 ± 4.84 (*n* = 3 independent primary culture), G93A:I-2 = 17.98 ± 3.12 (*n* = 5 independent primary culture), Q331K:CON = 42.76 ± 7.15 (*n* = 3 independent primary culture), Q331K:shPP1α = 38.71 ± 6.21 (*n* = 2 independent primary culture), Q331K:shPP1β = 21.69 ± 2.95 (*n* = 3 independent primary culture), Q331K:shPP1γ = 22.14 ± 1.44 (*n* = 3 independent primary culture), Q331K:I-2 = 14.45 ± 2.93 (*n* = 3 independent primary culture). Values are mean ± S.E.M. F(2,12) = 17.58, **P = 0.0012, ***P = 0.0007 for each CON group; F(4,14) = .101, ***P = 0.0007 for each GFP group; F(4,13) = 6.49, ^#^*P* = 0.0245 for G93A:CON versus G93A:shPP1γ, ^#^*P* = 0.0441 for G93A:CON versus G93A:I-2; F(4,9) = 7.405, ^#^*P* = 0.0433 for Q331K:CON versus Q331K: shPP1β, ^#^*P* = 0.0483 for Q331K:CON versus Q331K: shPP1γ, ^##^*P* = 0.0080 by one-way ANOVA followed by Tukey’s multiple comparisons test. **e** Analysis of total neurite length in (**a**). GFP:CON = 100 ± 8.70 (*n* = 57 GFP+ neurons), GFP:I-2 = 115.90 ± 9.90 (*n* = 22 GFP+ neuron), GFP: shPP1α=130.3 ± 18.51 (*n* = 9 GFP+ neuron), GFP:shPP1β=72.12 ± 8.25 (*n* = 16 GFP+ neuron), GFP:shPP1γ=60.66 ± 5.05 (*n* = 29 GFP+ neuron), G93A:CON = 61.61 ± 3.10 (*n* = 91 GFP+ neuron), G93A:I-2 = 106.3 ± 7.51 (*n* = 27 GFP+ neuron), G93A:shPP1α = 93.39 ± 13.05 (*n* = 21 GFP+ neuron), G93A:shPP1β = 57.56 ± 4.52 (*n* = 23 GFP+ neuron), G93A:shPP1γ = 88.06 ± 11.45 (*n* = 26 GFP+ neuron), Q331K:CON = 42.39 ± 3.47 (*n* = 25 GFP+ neuron), Q331K:I-2 = 122.2 ± 10.61 (*n* = 21 GFP+ neuron), Q331K:shPP1α=128.8 ± 17.3 (*n* = 23 GFP+ neuron), Q331K:shPP1β = 60.97 ± 6.15 (*n* = 27 GFP+ neuron), Q331K:shPP1γ = 94.81 ± 11.55 (*n* = 37 GFP+ neuron),. Values are mean ± S.E.M. F(2,170) = 19.98, ****P < 0.0001 for each CON group;F(4,128) = 7.415, ^#^*P* = 0.0106, ^##^*P* = 0.0025 for GFP:I-2 versus GFP:shPP1γ, ^##^*P* = 0.0057 for GFP:shPP1α versus GFP:shPP1γ; F(4,183)=9.91, ^#^*P* = 0.0235, ^#^*P* = 0.0089, ^###^*P* = 0.0008 for each G93A group; F(4,128) = 10.63, ^##^*P* = 0.0044, ^####^*P* < 0.0001 for each Q331K group by one-way ANOVA followed by Tukey’s multiple comparisons test. **f** Analysis of total axonal length in (**a**). GFP:CON = 100 ± 10.33 (*n* = 56 GFP+ neuron), GFP:I-2 = 119.4 ± 11.51 (*n* = 22 GFP+ neuron), GFP: shPP1α = 141.4 ± 24.44 (*n* = 9 GFP+ neuron), GFP:shPP1β = 73.71 ± 9.06 (*n* = 16 GFP+ neuron), GFP:shPP1γ = 59.58 ± 6.13 (*n* = 29 GFP+ neuron), G93A:CON = 54.08 ± 3.02 (*n* = 86 GFP+ neuron), G93A:I-2 = 80.63 ± 4.57 (*n* = 27 GFP+ neuron), G93A:shPP1α = 02.8 ± 15.10 (*n* = 21 GFP+ neuron), G93A:shPP1β = 58.73 ± 6.10 (*n* = 23 GFP+ neuron), G93A:shPP1γ = 95.06 ± 13.3 (*n* = 26 GFP+ neuron), Q331K:CON = 49.70 ± 7.46 (*n* = 28 GFP+ neuron), Q331K:I-2 = 131.7 ± 15.21 (*n* = 21 GFP+ neuron), Q331K:shPP1α = 145.8 ± 20.67 (*n* = 23 GFP+ neuron), Q331K:shPP1β = 60.02 ± 7.01 (*n* = 27 GFP+ neuron), Q331K:shPP1γ = 98.4 ± 12.79 (*n* = 37 GFP+ neuron). Values are mean ± S.E.M. F(2, 167) = 15.91, ***P = 0.0001, ****P < 0.0001 for each CON group; F(4,127) = 5.083, ^#^*P* = 0.0386 for each GF*P* group;F(4,178) = 9.565, ^#^*P* = 0.0356, ^###^*P* = 0.0002, ^####^*P* < 0.0001 for each G93A group;F(4,131) = 4.236, ^#^*P* = 0.0395, ^###^*P* = 0.0005, ^####^*P* < 0.0001 for each Q331K group by one-way ANOVA followed by Tukey’s multiple comparisons test.

To test whether PP1 inhibition is sufficient for the prevention of Drp1-dependent mitochondrial fission in the ALS model, we first examined whether PP1 inhibition prevented ALS-related mutant-dependent mitochondrial fragmentation. PP1 has three catalytic isoforms, α, β and γ, and I-2 can inhibit the action of all of them. Therefore, in order to explore their importance, the effects of specific shRNA against each isoform on mitochondrial fission were tested. Interestingly, the suppression of PP1α, γ, and I-2, but not PP1β, prevented the excessive fission of mitochondria in G93A and Q331K-overexpressed primary cortical neurons (Fig. 4a, b and Supplementary Fig. 3). Next, we tested whether suppression of PP1 prevented SOD1 G93A and TDP-43 Q331K-induced cell death. PP1 suppression by I-2 and shPP1γ reduced the number of active caspase-3-positive or pyknotic cells in the ALS-related mutant gene-overexpressed neurons (Fig. 4c, d). On the other hand, we found that the suppression of PP1α by shRNA significantly enhanced cell death. Yet the suppression of PP1 β and γ by shRNA significantly reduced the number of pyknotic cells in SOD1 G93A- or TDP-43 Q331K-overexpressed neurons (Fig. 4d). These results indicate that different PP1 isoforms may mediate different outcomes in ALS pathology via different substrate specificities. Furthermore, we also found that I-2 prevented the ALS-related mutant-induced retardation of axonal growth in zebrafish models (Fig. 4e, f). Expression of SOD1 G93A and/or TDP-43 Q331K reduced axonal growth, whereas I-2, shPP1α, and shPP1γ each restored axonal growth to a level equal to the control. Interestingly, this growth-restoring effect was not observed in Mdivi-1 treatments, suggesting that retardation of axonal growth was not entirely dependent on Drp1 activation and that PP1 may have additional targets for the progression of ALS-related axonal degeneration/growth retardation.

### Blockade of PP1 restores mitochondrial respiratory functions

**Fig. 5:**
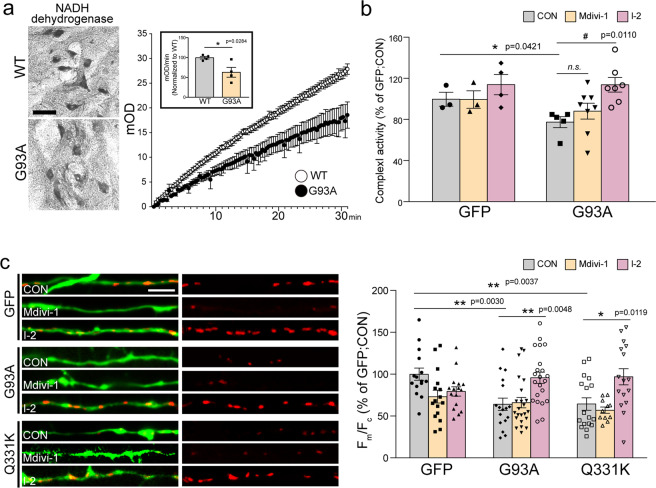
Effects of the blockade of PP1 on the complex I activity of mitochondria in the ALS model. **a** The histological image of NADH dehydrogenase activity and biochemical measurement of complex I activity on the MNs of the lumbar spinal cord in G93A and WT mice at 60 days of age. The inset graph shows the average of the complex I activity value. WT = 99.74 ± 3.83 (*n* = 4 mice), G93A = 62.9 ± 12.37 (*n* = 4 mice). Values are mean ± S.E.M. F(1,6) = 2.87, *P = 0.0284 by two-sided Student’s *t*-test. Scale bar, 100 μm. **b** The activity of mitochondrial complex I in primary cortical neurons cultured from WT and G93A mice following treatment with 25 µM Mdivi-1 or 80 nM I-2 for 72 h. WT:CON = 99.69 ± 6.81 (*n* = 3 independent primary culture), WT:Mdivi-1 = 99.37 ± 8.55 (*n* = 3 independent primary culture), WT:I-2 = 113.9 ± 9.12 (*n* = 4 independent primary culture), G93A:CON = 77.36 ± 5.33 (*n* = 5 independent primary culture), G93A:Mdivi-1 = 88.09 ± 7.70 (*n* = 8 independent primary culture), G93A:I-2 = 113.7 ± 7.09 (*n* = 7 independent primary culture). Values are mean ± S.E.M. F(1,6)=2.58, *P = 0.0421 for WT:CON versus G93A:CON by by two-sided Student’s *t*-test; F(2,17)=6.22, ^#^*P* = 0.0110 for G93A:CON versus G93A:I-2, n.s. = not significant group by one-way ANOVA followed by Tukey’s multiple comparisons test. **c** The representative images and measurement of MMP in GFP+ primary cortical neurons following mALS gene transfection with 25 µM Mdivi-1 or 80 nM I-2 treatment for 72 h. GFP:CON = 1 ± 0.07 (*n* = 15 GFP+ primary cortical neurons), GFP:Mdivi-1 = 0.73 ± 0.08 (*n* = 16 GFP+ primary cortical neurons), GFP:I-2 = 0.92 ± 0.06 (*n* = 16 GFP+ primary cortical neurons), G93A:CON = 0.66 ± 0.06 (*n* = 23 GFP+ primary cortical neurons), G93A:Mdivi-1 = 0.64 ± 0.07 (*n* = 17 GFP+ primary cortical neurons), G93A:I-2 = 0.96 ± 0.07 (*n* = 22 GFP+ primary cortical neurons), Q331K:CON = 0.64 ± 0.07 (*n* = 17 GFP+ primary cortical neurons), Q331K:Mdivi-1 = 0.57 ± 0.04 (*n* = 11 GFP+ primary cortical neurons), Q331K:I-2 = 0.97 ± 0.10 (*n* = 16 GFP+ primary cortical neurons). Values are mean ± S.E.M. F(2,52) = 7.529, **P = 0.0030 for GFP:CON versus G93A:CON, **P = 0.0037 for GFP:CON versus Q331K:GFP; F(2,44) = 3.86, *P = 0.0268 for GFP:CON versus GFP:Mdivi-1; F(2,59) = 7.184, **P = 0.0048 for G93A:CON versus G93A:I-2; F(2,41) = 6.87, *P = 0.0119 for Q331K:CON versus Q331K:I-2 by one-way ANOVA followed by Tukey’s multiple comparisons test. Scale bar, 20 μm.

Because the mitochondrial bioenergetics is expected to influence axonal growth, we tested whether PP1 activity is also associated with the alteration of mitochondrial respiratory complex activities (Fig. 5). It has been reported that complex I activity is primarily affected in G93A mice^39^, and we confirmed that NADH dehydrogenase, which represents mitochondrial complex I activity, was substantially reduced in the lumbar spinal cord of G93A mice, compared to WT (Fig. 5a). Next, we tested whether a blockade of PP1 or Drp1 activity rescued the impairment of mitochondrial function induced by the overexpression of SOD1 G93A or TDP-43 Q331K, which rapidly reduced complex I activity and mitochondrial membrane potential (MMP; Fig. 5b, c)^40–42^. However, I-2 effectively prevented the reduction of mitochondrial complex I activity and MMP in the mutant-overexpressed neurons, while Mdivi-1 treatment failed to rescue these functional impairments of the mitochondria. These results suggest that pathological PP1 activity impaired mitochondrial respiration via a Drp1-independent route as well as through Drp1-mediated mitochondrial impairments.

### Blockade of PP1 activity alleviates ALS pathology in vivo and in a human iPSC model

**Fig. 6:**
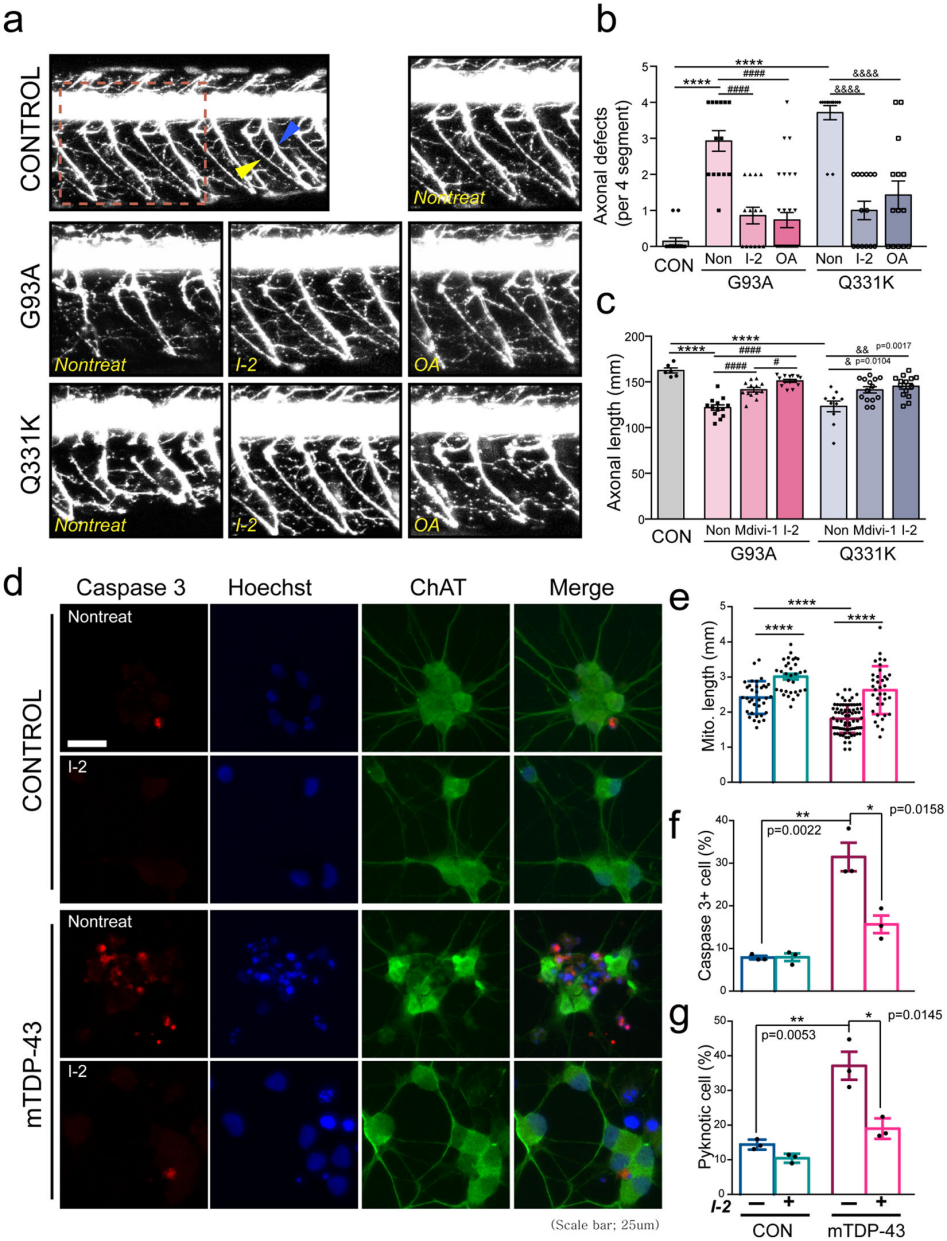
Effects of the suppression of PP1 in ALS-associated zebrafish and human iPS cell models. **a** Motor axons of zebrafish treated with 200 nM I-2 or 100 nM OA daily for 2 days following transient expression of G93A or Q331K mutations. The red dashed-line square indicates a magnified image of motor axons. The blue arrowhead indicates the ventral projection of the motor axon, and the yellow arrowhead indicates neuromuscular junction. **b** Analysis of axonal defects in (**a**). CON = 0.14 ± 0.36 (*n* = 14 zebrafish), G93A:Non = 2.93 ± 0.29 (*n* = 14 zebrafish), G93A:I-2 = 0.86 ± 0.23 (*n* = 14 zebrafish), G93A:OA = 0.73 ± 0.21 (*n* = 30 zebrafish), Q331K:Non = 3.71 ± 0.19 (*n* = 14 zebrafish), Q331K:I-2 = 1.00 ± 0.27 (*n* = 14 zebrafish), Q331K:OA = 1.43 ± 0.39 (*n* = 14 zebrafish). Values are mean ± S.E.M. F(2,39) = 81.83, ****P < 0.0001 for CON versus G93A:Non and CON versus Q331K:Non;F(2,55) = 21.78, ^####^*P* < 0.0001 for G93A:CON versus G93A:I-2 and G93A:CON versus G93A:OA; F(2,39) = 25.12, ^&&&&^*P* < 0.0001 for Q331K:CON versus Q331K:I-2 and Q331K:CON versus Q331K:OA by one-way ANOVA followed by Tukey’s multiple comparisons test. **c** Analysis of axonal length in (**a**). CON = 162.5 ± 2.77 (*n* = 6 zebrafish), G93A:Non = 122 ± 2.96 (*n* = 13 zebrafish), G93A:Mdivi-1 = 141.4 ± 2.45 (*n* = 13 zebrafish), G93A:I-2 = 151.1 ± 1.56 (*n* = 14 zebrafish), Q331K:Non = 23.3 ± 5.82 (*n* = 10 zebrafish), Q331K:Mdivi-1 = 141.3 ± 3.37 (*n* = 14 zebrafish), Q331K:I-2 = 145.3 ± 3.14 (*n* = 14 zebrafish). Values are mean ± S.E.M. F(2,26) = 21.27, ****P < 0.0001 for CON versus G93A:Non and CON versus Q331K:Non;F(2,37) = 39.83, ^####^*P* < 0.0001 for G93A:CON versus G93A:Mdivi-1 and G93A:CON versus G93A:I-2, ^#^*P* = 0.0156 for G93A:Mdivi-1 versus G93A:I-2;F(2,35 )= 7.755, ^&^*P* = 0.0104 for Q331K:CON versus Q331K:Mdivi-1, ^&&^*P* = 0.0017 for Q331K:CON versus Q331K:I-2 by one-way ANOVA followed by Tukey’s multiple comparisons test. **d** Immunostaining of cleaved caspase-3 and ChAT in 3-week-old MNs of control and mTDP-43 lines treated with 80 nM I-2 every other day for 2 weeks. Scale bar, 25 μm. **e** Measurement of mitochondrial length in 3-week-old MNs of control and mTDP-43 lines treated with 80 nM I-2 every other day for 2 weeks. CON:I-2(−) = 2.42 ± 0.07 (*n* = 38 ChAT+ neurons), CON:I-2(+) = 3.24 ± 0.13 (*n* = 37 ChAT+ neurons), mTDP-43:I-2(−) = 1.75 ± 0.06 (*n* = 38 ChAT+ neurons), mTDP-43:I-2(+) = 2.63 ± 0.11 (*n* = 36 ChAT+ neurons). Values are mean ± S.E.M. F(1,74) = 6.837, ****P < 0.0001 for CON:I-2(−) versus mTDP-43:I-2(−); F(1,73) = 5.538, ****P < 0.0001 for CON:I-2(−) versus CON:I-2(+); F(1,72) = 6.840, ****P < 0.0001 for mTDP-43:I-2(−) versus mTDP-43:I-2(+) for by two-sided Student’s *t*-test. **f** Quantification of cleaved caspase-3^+^ cell in human iPS-derived ChAT^+^ neuron in (**d**). CON:I-2(−) = 7.86 ± 0.42 (*n* = 3 iPS cell lines), CON:I-2(+) = 7.96 ± 0.89 (*n* = 3 iPS cell lines), mTDP-43:I-2(−) = 31.47 ± 3.34 (*n* = 3 iPS cell lines), mTDP-43:I-2(+) = 15.67 ± 2.06 (*n* = 3 iPS cell lines). Values are mean ± S.E.M. F(1,4) = 7.01, **P = 0.0022 for CON:I-2(−) versus mTDP-43:I-2(−); F(1,4) = 4.03, *P = 0.0158 for mTDP-43:I-2(−) versus mTDP-43:I-2(+) for by two-sided Student’s *t*-test. **g** Quantification of pyknotic cell in ChAT^+^ neuron in (**d**). Values are mean ± S.E.M. (*N* = 3 lines in CON and mTDP-43; *p < 0.05, **p < 0.01 in two-sided Student’s *t*-test). CON:I-2(−) = 14.37 ± 0.84 (*n* = 3 iPS cell lines), CON:I-2(+) = 10.43 ± 0.75 (*n* = 3 iPS cell lines), mTDP-43:I-2(−) = 7.11 ± 4.04 (*n* = 3 iPS cell lines), mTDP-43:I-2(+) = 18.99 ± 1.71 (*n* = 3 iPS cell lines). Values are mean ± S.E.M. F(1,4) = 5.514, **P = 0.0053 for CON:I-2(−) versus mTDP-43:I-2(−); F(1,4) = 4.131, *P = 0.0145 for mTDP-43:I**-**2(−) versus mTDP-43:I-2(+) for by two-sided Student’s *t*-test.

Next, we tested whether PP1 inhibition can effectively prevent ALS-related neurodegeneration in a zebrafish model. PP1 inhibition by treatment with I-2 or okadaic acid (OA) prevented the axonal defects induced by SOD1 G93A or TDP-43 Q331K mutations (Fig. 6a, b). Interestingly, I-2 restored the motor axonal length of mutant-expressing MNs to a level equivalent to the control, which was significantly superior to the effects of axonal elongation by the Mdivi-1 treatment (Fig. 6c). Finally, we also tested whether these preventive effects of I-2 could be observed in a human iPSC-derived MN model in vitro. First, we differentiated multiple iPS cell lines from controls and TDP43 mutants (mTDP-43;A90V, Q343R, M337V)^43^ into spinal MNs as described^21^. PP1 suppression induced by I-2 treatment over 2 weeks inhibited mitochondrial fragmentation, although in the periods between administrations of I-2, the mTDP group exhibited noticeable mitochondrial shortening (Fig. 6a). As anticipated, the number of active caspase-3-positive neurons and pyknotic cells was lower in the I-2-treated mTDP group, suggesting that inhibition of PP1 by I-2 suppresses neuronal toxicity in several ALS-associated models. The prevention of pathological PP1 activity could, therefore, be a viable therapeutic target.

## Discussion

**Fig. 7:**
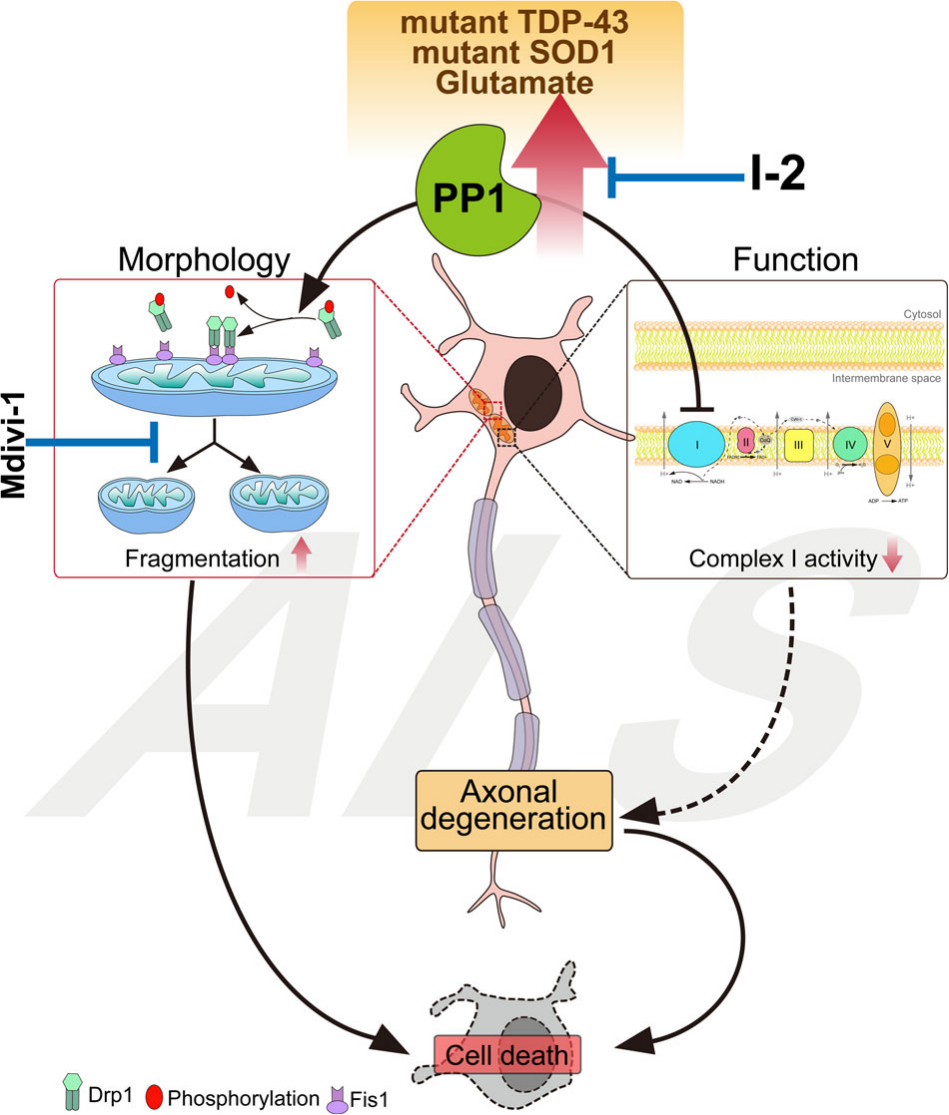
Hypothetical schematic diagram: abnormal promotion of PP1 activity in the MNs of ALS and dephosphorylation of the S616 site of Drp1, resulting in the induction of mitochondrial fragmentation. Also, abnormal PP1 activity decreases the mitochondrial complex I activity, leading to axonal degeneration. Thus, the blockade of PP1 activity reduces the ALS-induced mitochondrial fission and improves mitochondrial functions such as the complex I activity of the mitochondrial electron transport chain and membrane potential, resulting in improvement of axonal impairment. Therefore, inhibition of excessive PP1 activity may promote MN survival by improving mitochondrial function.

Recent large-scale genetic analyses have revealed that genes mediating many different biological cascades are associated with ALS, suggesting that many different pathways should merge into a common ‘hub’ cascade to exhibit common symptoms such as MN death, axonal degeneration, and mitochondrial defects^44,45^. In this study, we identified that dephosphorylation of Drp1 is mediated by pathological activation of PP1 induced by SOD1 G93A or TDP-43 Q331K overexpression in cellular models, and in mouse models with the G93A mutation. Blocking the PP1-Drp1 cascades effectively prevented mitochondrial defects and subsequent neurodegeneration in various ALS models. The gain-of-function mutation of SOD1 is proposed to impair protein misfolding-mediated clearance pathways, including ubiquitin-dependent proteolysis, protein aggregations, and autophagy, in addition to causing mitochondrial dysfunction^46–48^. On the other hand, TDP-43 is an RNA-binding protein that may trigger defects in mRNA processing, with multiple consequences including defective nucleocytoplasmic transport^49^, mitochondrial deficits, and abnormalities in mitophagy^50,51^. Given that these different causative mutations commonly compromise mitochondrial function, which can be prevented by the blockade of PP1-Drp1 cascades, these form an important common biological hub for ALS pathology, and may be important therapeutic targets against the multiple pathological features of ALS. Further analysis with different ALS models caused by other types of mutation is necessary to expand upon this intriguing notion (Fig. 7).

Mitochondrial defects have been considered important mediators in the progression of multiple neurodegenerative diseases, including ALS. In ALS models, the mitochondria are hyper-fragmented, and their axonal transport, ATP synthesis, and mitophagic removal are greatly affected^28,52,53^. Drp1 has been proposed as a key player in this mitochondria-related ALS pathology because several causative gene mutations associated with ALS promote Drp1 activity^36,54–57^. The induction of Drp1 activity is apt to promote cell death signaling via multiple mediating processes^39,58^. Thus, the inhibition of Drp1 led to the exhibition of therapeutic effects in the G93A mouse model^59^. Our current observations further confirmed this notion in SOD1 mutations and extended it to include ALS models with the TDP-43 mutation. Notably, we observed the dephosphorylation of Drp1 S616 sites in several ALS models, and Drp1 S616 has been widely considered as an activating phosphorylation site^30^. However, we previously found that phosphorylation of Drp1 S616 by cyclin-dependent kinase 5 (CDK5) inhibited the translocation of Drp1 to the mitochondria in neurons, resulting in reduced mitochondrial fission^36,60^. Given that CDK5 is specifically expressed in post-mitotic neurons, this cascade appeared to be specific to neurons at that particular stage. In this respect, the dephosphorylation of Drp1 S616 in the neurons of ALS models appears to promote Drp1 activation. We found that a blockade of dephosphorylation prevented Drp1-related ALS symptoms, which supports our current hypothesis.

While Drp1 inhibition proved to be clearly beneficial, there were nonetheless several reasons for us to seek upstream targets, such as PP1, for ALS therapy. Previously, we observed that a blockade of Drp1 activity in normally developing chick MNs resulted in an increase in programmed cell death and the impairment of axonal growth^61^. Considering that mitochondrial fusion and fission are essential events for cellular homeostasis^28,30,53^, there is a narrow range in which mitochondrial dynamics must be maintained for optimal survival. In concurrence with our previous observations in developing MNs, we also found that the suppression of Drp1 activation had little benefit for axonal growth. It should also be noted that Drp1 performs many functions in addition to mitochondrial fission. Drp1 can interact with many different proteins localized at different subcellular locations, thereby being involved in many biological processes such as peroxisomal fragmentation, vesicle endocytosis/recycling, MMP changes, and cytoskeletal remodeling^29,62^. Indeed, restoration of Drp1 activity to a normal physiological level in the long-term may be difficult to achieve, meaning that alternative robust and druggable targets for ALS therapy are highly desirable.

Here we identified PP1 as an upstream phosphatase responsible for the dephosphorylation of Drp1 and tested whether regulation of PP1 activity is beneficial for the suppression of ALS-related pathological consequences. PP1 is a serine/threonine phosphatase and exists as a cellular holoenzyme composed of catalytic and regulatory subunits^38,63,64^. It is required for many cellular functions, including cell division, apoptosis, cytoskeletal organization, protein synthesis, and metabolism^38^. Moreover, it has been reported that PP1 activity is associated with several neurodegenerative diseases. For instance, the dephosphorylation of mutant leucine-rich repeat kinase 2 (LRRK2) by PP1 is increased in Parkinson’s disease^65^. Previous studies reported that, in Alzheimer’s disease, tau phosphorylation was induced by GSK3β, which is activated by PP1^66,67^, and I-2 prevented amyloid-β oligomer (AβO)-induced defects in brain-derived neurotrophic factor (BDNF)-containing vesicle transport^67^. In this study, we found that PP1 activity was increased in the SOD1 G93A ALS mouse model before the onset of the disease, and the blockade of PP1 activity prevented mitochondrial defects. Importantly, the PP1 blockade prevented axonal degeneration and restored the complex I activity of the mitochondria in the ALS model, results that were not achieved by the inhibition of Drp1 in our model. We found that the different PP1 isoforms may mediate different consequences of ALS-related pathologies because selective inhibition of each isoform resulted in different outcomes with respect to mitochondrial fragmentation, axonal growth, and cell survival. Das et al. reported that Sephin1, a novel inhibitor of PP1 and PPP1R15α (GADD34) binding, prevented the aggregation of mutant SOD1 G93A protein and MN death in an ALS model, improving motor deficiency in SOD1 G93A mice^68^. They proposed that Sephin1 inhibited the phosphorylation of eukaryotic initiation factor 2 (eIF2α) without affecting PP1 catalytic activity. On the other hand, we observed that the inhibition of PP1 activity improved ALS-related pathologies and failed to observe any dephosphorylation of eIF2 α induced by I-2 treatment (Supplementary Fig. 4). Therefore, PP1 may affect the propagation of ALS-related pathologies in catalytic activity-dependent or -independent manners.

While PP1 appears to affect ALS pathology via Drp1 dephosphorylation, it must also have other substrates. For instance, mitochondrial complex I, which is composed of 44 subunits^69^, is a pacemaker of the mitochondrial respiratory system^70,71^. Thus, impairment of complex I is often reported in ALS models^1,2^. Complex I deficiency represents severe defects within the mitochondrial system and induces ALS *via* impairment of mitochondrial metabolism^72–74^. Some subunits in complex I, which are closely linked to its activity, are regulated by (de)phosphorylation^63,75–77^. Therefore, although we cannot completely rule out the possibility that the pathological effect of PP1 activation is also mediated by Drp1, we favor the idea that pathological induction of PP1 activity may impair other cellular processes, including mitochondrial complex activity, independently of Drp1, via dephosphorylation of its target protein(s). Unbiased identification of PP1 targets and the further assessment of the precise molecular targets of PP1 should be addressed in the future to clarify this and related issues.

## Supplementary information

Supplementary Figure 1

Supplementary Figure 2

Supplementary Figure 3

Supplementary Figure 4

Supplementary figure legends (Unmarked)

## References

[1] Wijesekera LC, Leigh PN (2009). Amyotrophic lateral sclerosis. Orphanet J. Rare Dis..

[2] Song W, Song Y, Kincaid B, Bossy B, Bossy-Wetzel E (2013). Mutant SOD1G93A triggers mitochondrial fragmentation in spinal cord motor neurons: neuroprotection by SIRT3 and PGC-1α. Neurobiol. Dis..

[3] Deng H, Gao K, Jankovic J (2014). The role of FUS gene variants in neurodegenerative diseases. Nat. Rev. Neurol..

[4] Cooper-Knock J (2012). Clinico-pathological features in amyotrophic lateral sclerosis with expansions in C9ORF72. Brain.

[5] DeJesus-Hernandez M (2011). Expanded GGGGCC hexanucleotide repeat in noncoding region of C9ORF72 causes chromosome 9p-linked FTD and ALS. Neuron.

[6] Harms MB, Baloh RH (2013). Clinical neurogenetics: amyotrophic lateral sclerosis. Neurol. Clin..

[7] Byrne S (2012). Cognitive and clinical characteristics of patients with amyotrophic lateral sclerosis carrying a C9orf72 repeat expansion: a population-based cohort study. Lancet Neurol..

[8] Majounie E (2012). Frequency of the C9orf72 hexanucleotide repeat expansion in patients with amyotrophic lateral sclerosis and frontotemporal dementia: a cross-sectional study. Lancet Neurol..

[9] Parone PA (2013). Enhancing mitochondrial calcium buffering capacity reduces aggregation of misfolded SOD1 and motor neuron cell death without extending survival in mouse models of inherited amyotrophic lateral sclerosis. J. Neurosci..

[10] Shaw PJ, Ince PG (1997). Glutamate, excitotoxicity and amyotrophic lateral sclerosis. J. Neurol..

[11] Zürcher NR (2015). Increased in vivo glial activation in patients with amyotrophic lateral sclerosis: assessed with [(11)C]-PBR28. Neuroimage Clin..

[12] Tank EM (2018). Abnormal RNA stability in amyotrophic lateral sclerosis. Nat. Commun..

[13] Faes L, Callewaert G (2011). Mitochondrial dysfunction in familial amyotrophic lateral sclerosis. J. Bioenerg. Biomembr..

[14] Dupuis L (2004). Mitochondria in amyotrophic lateral sclerosis: a trigger and a target. Neurodegener. Dis..

[15] Miller RG, Mitchell JD, Moore DH (2012). Riluzole for amyotrophic lateral sclerosis (ALS)/motor neuron disease (MND). Cochrane Database Syst. Rev..

[16] Sawada H (2019). Considerations for pharmacotherapy use in patients with amyotrophic lateral sclerosis: the earlier it starts, the better the results. Expert Opin. Pharmacother..

[17] Abe K (2014). Confirmatory double-blind, parallel-group, placebo-controlled study of efficacy and safety of edaravone (MCI-186) in amyotrophic lateral sclerosis patients. Amyotroph. Lateral Scler. Frontotemporal Degener..

[18] Smith EF, Shaw PJ, De Vos KJ (2019). The role of mitochondria in amyotrophic lateral sclerosis. Neurosci. Lett..

[19] Sasaki S, Iwata M (1996). Impairment of fast axonal transport in the proximal axons of anterior horn neurons in amyotrophic lateral sclerosis. Neurology.

[20] Manfredi G, Xu Z (2005). Mitochondrial dysfunction and its role in motor neuron degeneration in ALS. Mitochondrion.

[21] Sasaki S, Iwata M (1996). Ultrastructural study of synapses in the anterior horn neurons of patients with amyotrophic lateral sclerosis. Neurosci. Lett..

[22] Kong J, Xu Z (1998). Massive mitochondrial degeneration in motor neurons triggers the onset of amyotrophic lateral sclerosis in mice expressing a mutant SOD1. J. Neurosci..

[23] Lopez-Gonzalez R (2016). Poly(GR) in C9ORF72-Related ALS/FTD Compromises Mitochondrial Function and Increases Oxidative Stress and DNA Damage in iPSC-Derived Motor. Neurons Neuron.

[24] Choi SY (2019). C9ORF72-ALS/FTD-associated poly(GR) binds Atp5a1 and compromises mitochondrial function in vivo. Nat. Neurosci..

[25] Carriedo SG, Yin HZ, Weiss JH (1996). Motor neurons are selectively vulnerable to AMPA/kainate receptor-mediated injury in vitro. J. Neurosci..

[26] Saxena S, Caroni P (2011). Selective neuronal vulnerability in neurodegenerative diseases: from stressor thresholds to degeneration. Neuron.

[27] Kruman II, Pedersen WA, Springer JE, Mattson MP (1999). ALS-linked Cu/Zn-SOD mutation increases vulnerability of motor neurons to excitotoxicity by a mechanism involving increased oxidative stress and perturbed calcium homeostasis. Exp. Neurol..

[28] Bereiter-Hahn J, Jendrach M (2010). Mitochondrial dynamics. Int. Rev. Cell Mol. Biol..

[29] Cho HM (2019). Drp1-Zip1 Interaction regulates mitochondrial quality surveillance system. Mol. Cell.

[30] Cho B, Choi SY, Cho HM, Kim HJ, Sun W (2013). Physiological and pathological significance of dynamin-related protein 1 (drp1)-dependent mitochondrial fission in the nervous system. Exp. Neurobiol..

[31] Liu W (2013). Mitochondrial fusion and fission proteins expression dynamically change in a murine model of amyotrophic lateral sclerosis. Curr. Neurovasc Res..

[32] Stevens JC (2010). Modification of superoxide dismutase 1 (SOD1) properties by a GFP tag-implications for research into amyotrophic lateral sclerosis (ALS). PLoS ONE.

[33] Shin J, Park H-C, Topczewska JM, Mawdsley DJ, Appel B (2003). Neural cell fate analysis in zebrafish using olig2 BAC transgenics. Methods Cell Sci..

[34] Hou H (2013). Synaptic NMDA receptor stimulation activates PP1 by inhibiting its phosphorylation by Cdk5. J. Cell Biol..

[35] De Paepe B, De Bleecker JL, Van Coster R (2009). Histochemical methods for the diagnosis of mitochondrial diseases. Curr. Protoc. Hum. Genet..

[36] Cho B (2014). CDK5-dependent inhibitory phosphorylation of Drp1 during neuronal maturation. Exp. Mol. Med..

[37] Dohadwala M (1994). Phosphorylation and inactivation of protein phosphatase 1 by cyclin-dependent kinases. Proc. Natl Acad. Sci. USA.

[38] Korrodi-Gregório L, Esteves SLC, Fardilha M (2014). Protein phosphatase 1 catalytic isoforms: specificity toward interacting proteins. Transl. Res..

[39] Coussee E (2011). G37R SOD1 mutant alters mitochondrial complex I activity, Ca(2+) uptake and ATP production. Cell Calcium.

[40] Salvatori I (2018). Differential toxicity of TAR DNA‐binding protein 43 isoforms depends on their submitochondrial localization in neuronal cells. J. Neurochemistry.

[41] Wang W (2016). The inhibition of TDP-43 mitochondrial localization blocks its neuronal toxicity. Nat. Med..

[42] Davis SA (2018). TDP-43 interacts with mitochondrial proteins critical for mitophagy and mitochondrial dynamics. Neurosci. Lett..

[43] Egawa N (2012). Drug screening for ALS using patient-specific induced pluripotent stem cells. Sci. Transl. Med..

[44] Maniatis S (2019). Spatiotemporal dynamics of molecular pathology in amyotrophic lateral sclerosis. Science.

[45] Sun S (2015). Translational profiling identifies a cascade of damage initiated in motor neurons and spreading to glia in mutant SOD1-mediated ALS. Proc. Natl Acad. Sci. U. S. A..

[46] Rotunno MS, Bosco DA (2013). An emerging role for misfolded wild-type SOD1 in sporadic ALS pathogenesis. Front Cell Neurosci..

[47] Ramesh N, Pandey UB (2017). Autophagy dysregulation in ALS: when protein aggregates get out of hand. Front Mol. Neurosci..

[48] Yung C, Sha D, Li L, Chin L-S (2016). Parkin protects against misfolded SOD1 toxicity by promoting its aggresome formation and autophagic clearance. Mol. Neurobiol..

[49] Chou C-C (2018). TDP-43 pathology disrupts nuclear pore complexes and nucleocytoplasmic transport in ALS/FTD. Nat. Neurosci..

[50] Wang P (2019). TDP-43 induces mitochondrial damage and activates the mitochondrial unfolded protein response. PLoS Genet..

[51] Prasad A, Bharathi V, Sivalingam V, Girdhar A, Patel BK (2019). Molecular mechanisms of TDP-43 misfolding and pathology in amyotrophic lateral sclerosis. Front Mol. Neurosci..

[52] Knott AB, Bossy-Wetzel E (2008). Impairing the mitochondrial fission and fusion balance: a new mechanism of neurodegeneration. Ann. N. Y. Acad. Sci..

[53] Otera H, Mihara K (2012). Mitochondrial dynamics: functional link with apoptosis. Int J. Cell Biol..

[54] Saez-Atienzar S (2014). The LRRK2 inhibitor GSK2578215A induces protective autophagy in SH-SY5Y cells: involvement of Drp-1-mediated mitochondrial fission and mitochondrial-derived ROS signaling. Cell Death Dis..

[55] Wang H (2011). Parkin ubiquitinates Drp1 for proteasome-dependent degradation: implication of dysregulated mitochondrial dynamics in Parkinson disease. J. Biol. Chem..

[56] Manczak M, Reddy PH (2012). Abnormal interaction between the mitochondrial fission protein Drp1 and hyperphosphorylated tau in Alzheimer’s disease neurons: implications for mitochondrial dysfunction and neuronal damage. Hum. Mol. Genet..

[57] Otera H, Mihara K (2011). Discovery of the membrane receptor for mitochondrial fission GTPase Drp1. Small GTPases.

[58] Hoppins S (2014). The regulation of mitochondrial dynamics. Curr. Opin. Cell Biol..

[59] Joshi AU (2018). Inhibition of Drp1/Fis1 interaction slows progression of amyotrophic lateral sclerosis. EMBO Mol. Med..

[60] Cribbs JT, Strack S (2007). Reversible phosphorylation of Drp1 by cyclic AMP-dependent protein kinase and calcineurin regulates mitochondrial fission and cell death. EMBO Rep..

[61] Choi SY (2013). Drp1-mediated mitochondrial dynamics and survival of developing chick motoneurons during the period of normal programmed cell death. FASEB J..

[62] Li H (2013). A Bcl-xL-Drp1 complex regulates synaptic vesicle membrane dynamics during endocytosis. Nat. Cell Biol..

[63] Choy MS, Page R, Peti W (2012). Regulation of protein phosphatase 1 by intrinsically disordered proteins. Biochem. Soc. Trans..

[64] Peti W, Nairn AC, Page R (2013). Structural basis for protein phosphatase 1 regulation and specificity. FEBS J..

[65] Lobbestael E (2013). Identification of protein phosphatase 1 as a regulator of the LRRK2 phosphorylation cycle. Biochem. J..

[66] Braithwaite SP, Stock JB, Lombroso PJ, Nairn AC (2012). Protein phosphatases and Alzheimer’s disease. Prog. Mol. Biol. Transl. Sci..

[67] Ramser EM (2013). Amyloid-β oligomers induce tau-independent disruption of BDNF axonal transport via calcineurin activation in cultured hippocampal neurons. Mol. Biol. Cell.

[68] Das I (2015). Preventing proteostasis diseases by selective inhibition of a phosphatase regulatory subunit. Science.

[69] Carroll J (2006). Bovine complex I is a complex of 45 different subunits. J. Biol. Chem..

[70] Hüttemann M, Lee I, Samavati L, Yu H, Doan JW (2007). Regulation of mitochondrial oxidative phosphorylation through cell signaling. Biochim. Biophys. Acta.

[71] Remacle C, Barbieri MR, Cardol P, Hamel PP (2008). Eukaryotic complex I: functional diversity and experimental systems to unravel the assembly process. Mol. Genet. Genomics.

[72] Smeitink J, van den Heuvel L, DiMauro S (2001). The genetics and pathology of oxidative phosphorylation. Nat. Rev. Genet..

[73] Kruse SE (2008). Mice with mitochondrial complex I deficiency develop a fatal encephalomyopathy. Cell Metab..

[74] Petruzzella V, Papa S (2002). Mutations in human nuclear genes encoding for subunits of mitochondrial respiratory complex I: the NDUFS4 gene. Gene.

[75] Palmisano G, Sardanelli AM, Signorile A, Papa S, Larsen MR (2007). The phosphorylation pattern of bovine heart complex I subunits. Proteomics.

[76] Pocsfalvi G (2007). Phosphorylation of B14.5a subunit from bovine heart complex I identified by titanium dioxide selective enrichment and shotgun proteomics. Mol. Cell Proteom..

[77] Schilling B (2005). Mass spectrometric identification of a novel phosphorylation site in subunit NDUFA10 of bovine mitochondrial complex I. FEBS Lett..

