# Supplementary figures and images for: Prevention of mitochondrial impairment by inhibition of protein phosphatase 1 activity in amyotrophic lateral sclerosis
Source: Cell Death Dis. 2020 Oct 21;11(10):888. doi: 10.1038/s41419-020-03102-8 (PMC7578657; doi:10.1038/s41419-020-03102-8)

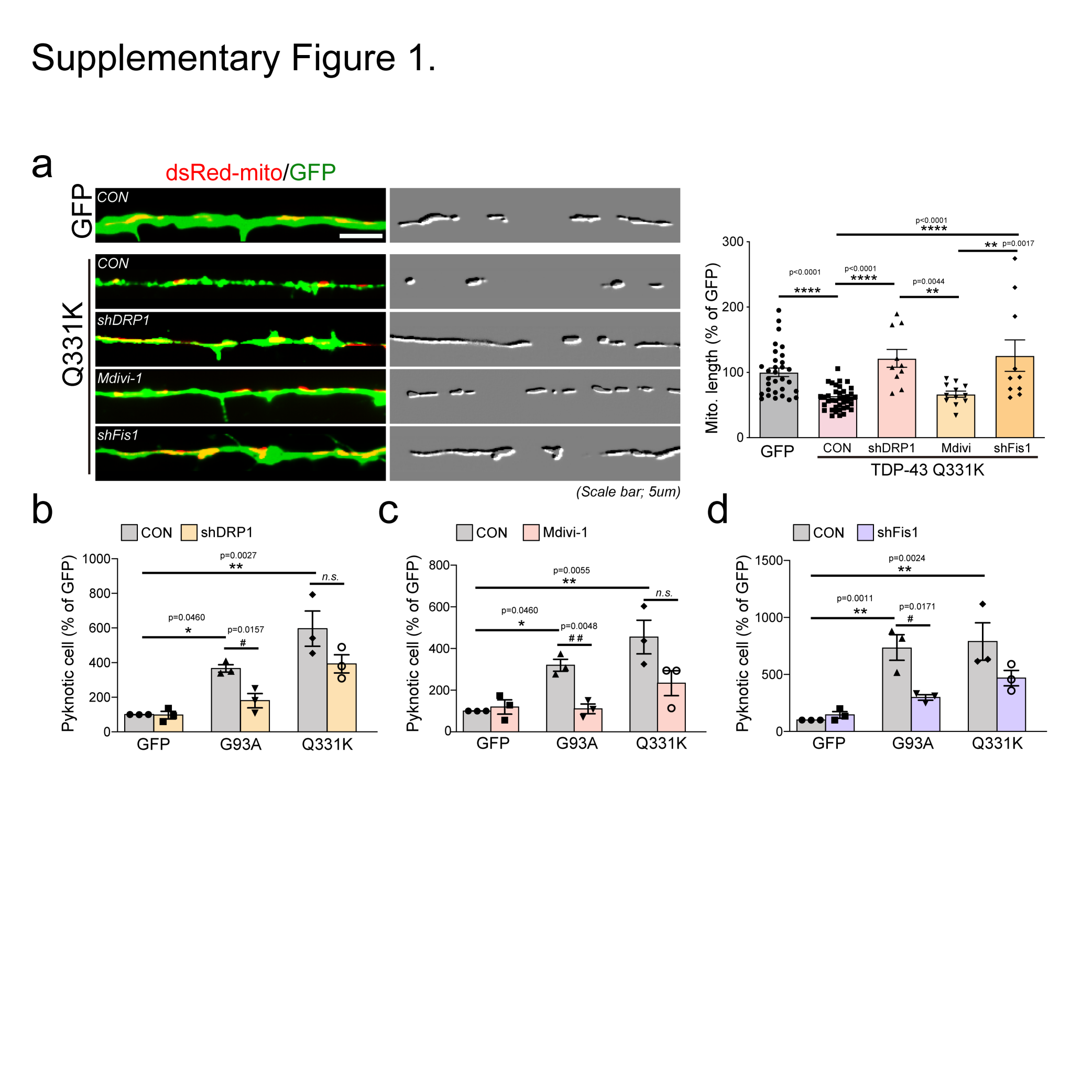

Supplement: Supplementary file 1 — Supplementary Figure 1 [file 41419_2020_3102_MOESM1_ESM.tif]

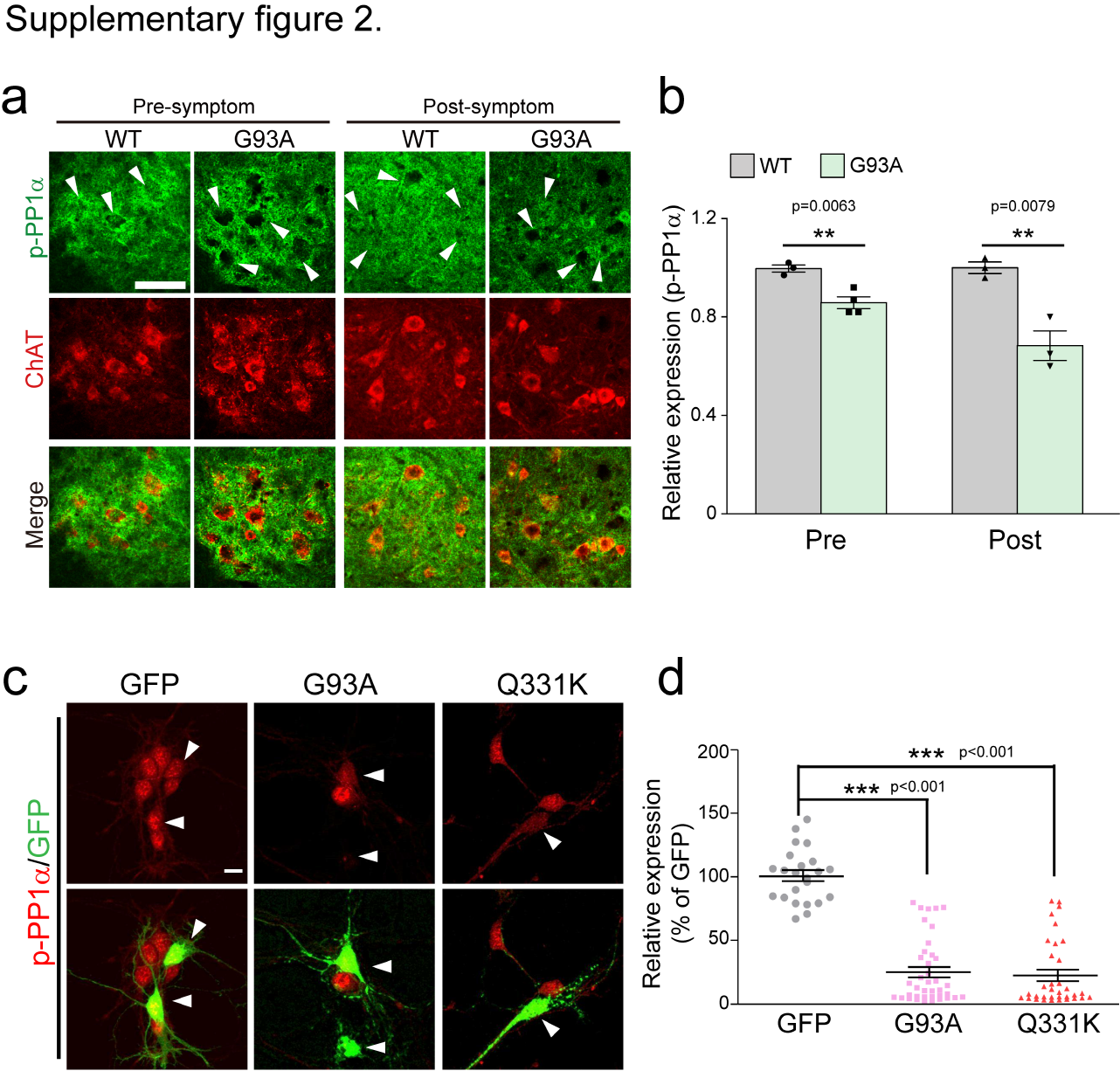

Supplement: Supplementary file 2 — Supplementary Figure 2 [file 41419_2020_3102_MOESM2_ESM.tif]

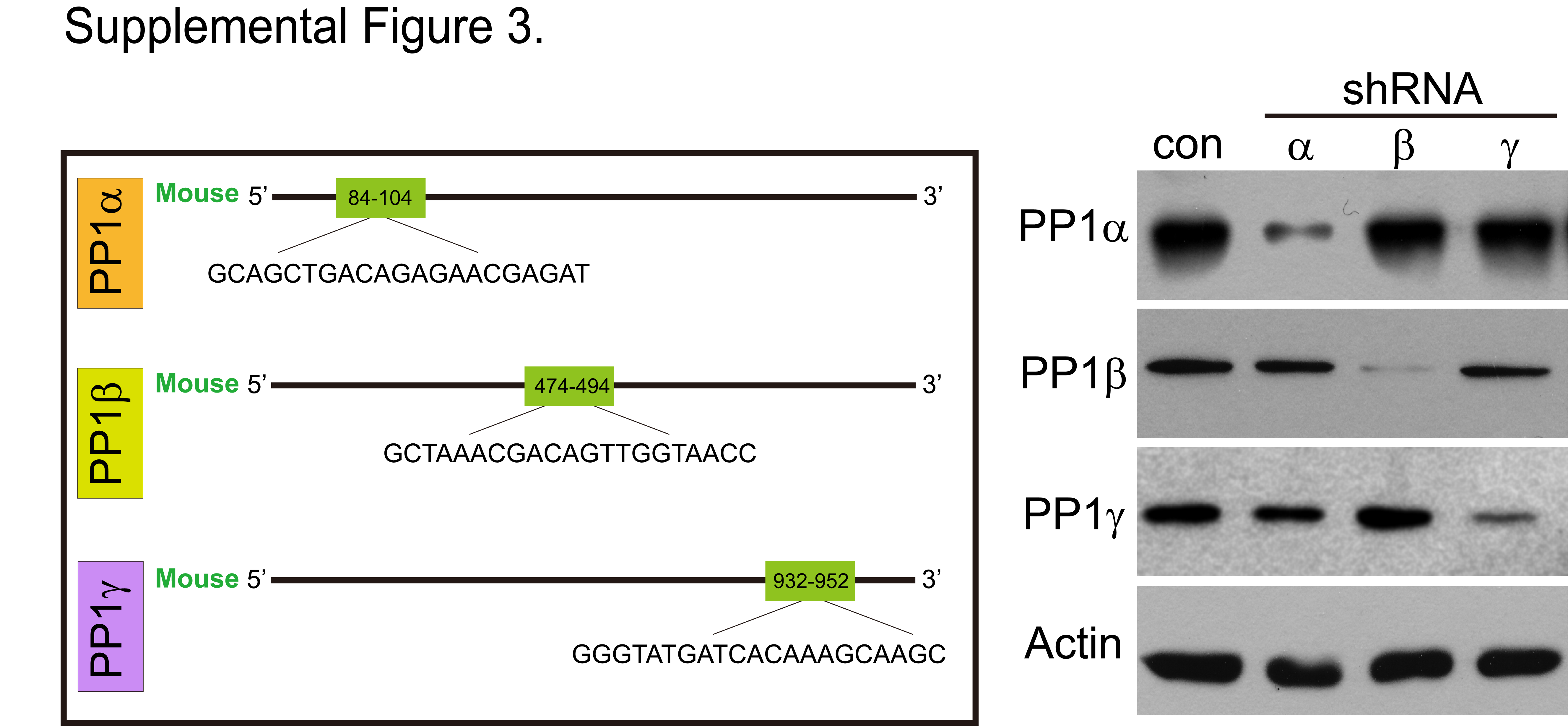

Supplement: Supplementary file 3 — Supplementary Figure 3 [file 41419_2020_3102_MOESM3_ESM.tif]

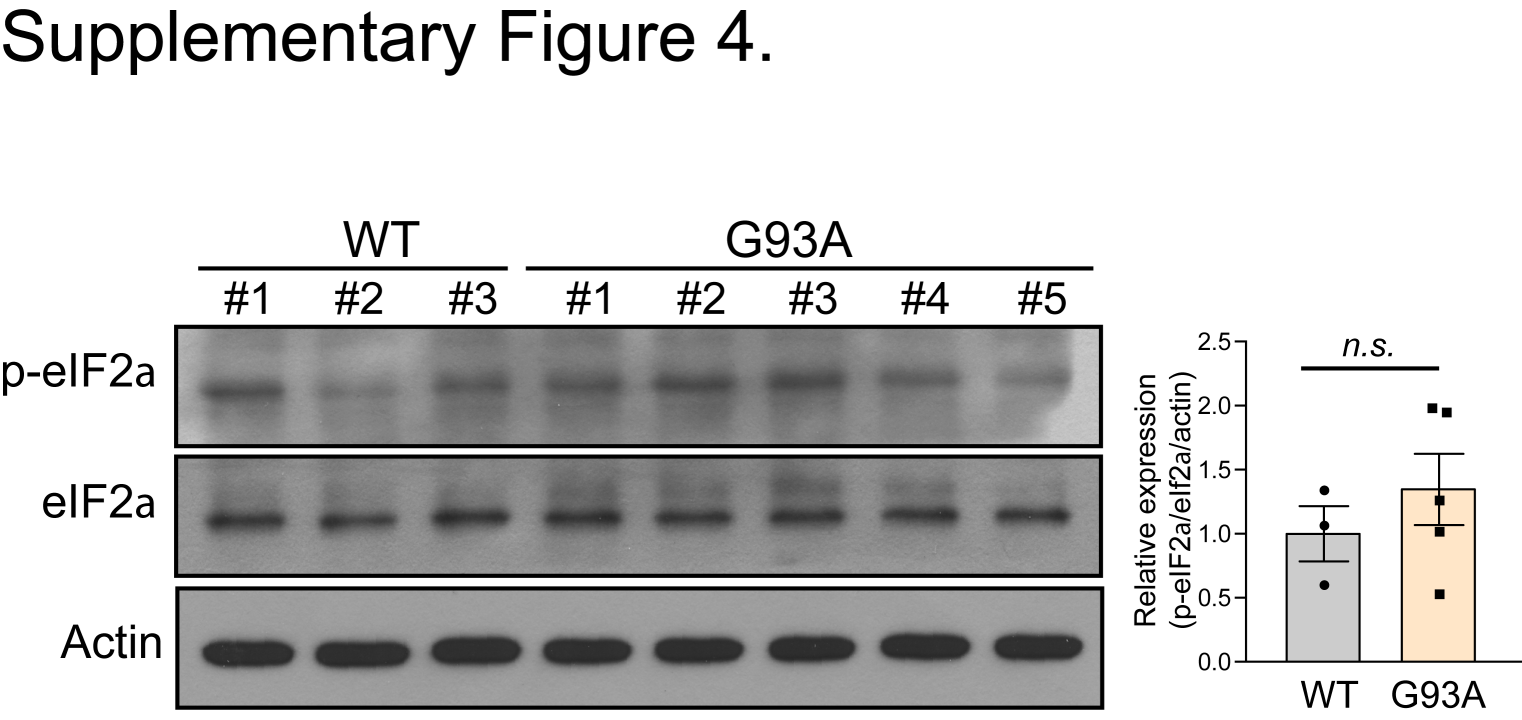

Supplement: Supplementary file 4 — Supplementary Figure 4 [file 41419_2020_3102_MOESM4_ESM.tif]
