# Supplementary material for: Prevention of mitochondrial impairment by inhibition of protein phosphatase 1 activity in amyotrophic lateral sclerosis
Source: Cell Death Dis. 2020 Oct 21;11(10):888. doi: 10.1038/s41419-020-03102-8 (PMC7578657; doi:10.1038/s41419-020-03102-8)
Supplement: Supplementary file 5 — Supplementary figure legends (Unmarked) [file 41419_2020_3102_MOESM5_ESM.docx]

**Supplementary figure legends**

**Supplementary Fig 1.** Effects of the blockade of Drp1 activity on the TDP-43 Q331K mutant-derived mitochondria. Three days after the co-transfection of shRNAs or treatment of 25 μM Mdivi-1 with a Q331K clone, analysis of mitochondrial length (**a**) and active caspase-3-positive neurons (**b-d**). Mitochondrial images have been embossed. Scale bar, 5μm. GFP=100.1±6.54 (n=31 GFP+ neurons), TDP-43 Q331K:CON=60.01±2.70 (n=39 GFP+ neurons), TDP-43 Q331K:shDRP1=121.5±13.70 (n=10 GFP+ neurons), TDP-43 Q331K:Mdivi=96.53±12.53 (n=13 GFP+ neurons), TDP-43 Q331K:shFis1=125.7±24.05 (n=10 GFP+ neurons). Values are mean **±**S.E.M. F(1,68)=6.115, ***P*<0.0001 for GFP versus TDP-43 Q331K:CON by two-sided Student’s *t*-test; F(3,68)=12.17, ***P*=0.0044 for TDP-43 Q331K:shDRP1 versus TDP-43 Q331K:Mdivi, ***P*=0.0017 for TDP-43 Q331K:Mdivi versus TDP-43 Q331K:shFis1, *****P*<0.0001 by one-way ANOVA with Tukey’s post hoc analysis for multiple comparisons in **a**. GFP:CON=100±0.02 (n=3 independent primary cultures), GFP:shDRP1=96.71±22.21 (n=3 independent primary cultures), G93A:CON=366.3±21.43 (n=3 independent primary cultures), G93A:shDRP1=180±40.87 (n=3 independent primary cultures), Q331K:CON=596±101.8 (n=3 independent primary cultures), Q331K:shDRP1=393±52.44 (n=3 independent primary cultures). Values are mean **±**S.E.M. F(2,6)=12.79, **P*=0.0460, ***P*=0.0027 by one-way ANOVA with Tukey’s post hoc analysis for multiple comparisons; F(1,4)=4.036, #*P*=0.0157, *n.s.*=not significant by two-sided Student’s *t*-test in **b**. GFP:CON=99.93±0.07 (n=3 independent primary cultures), GFP:Mdivi-1=119.3±34.13 (n=3 independent primary cultures), G93A:CON=319.6±28.71 (n=3 independent primary cultures), G93A:Mdivi-1=110±23.34 (n=3 independent primary cultures), Q331K:CON=454.8±80.79 (n=3 independent primary cultures), Q331K: Mdivi-1=233.7±60.09 (n=3 independent primary cultures). Values are mean **±**S.E.M. F(2,6)=13.09, **P*=0.0460, ***P*=0.0055 by one-way ANOVA with Tukey’s post hoc analysis for multiple comparisons; F(1,4)=5.663, #*P*=0.0048, *n.s.*=not significant by two-sided Student’s *t*-test in **c**. GFP:CON=100.1±0.04 (n=3 independent primary cultures), GFP:shFis1=145.1±29.33 (n=3 independent primary cultures), G93A:CON=739.9±109.6 (n=3 independent primary cultures), G93A: shFis1=298.3±24.3 (n=3 independent primary cultures), Q331K:CON=649.5±25.81 (n=3 independent primary cultures), Q331K:shFis1=468.4±67.43 (n=3 independent primary cultures). Values are mean **±**S.E.M. F(2,6)=28.36, ***P*=0.0011 for GFP:CON versus G93A:CON, ***P*=0.0024 for GFP:CON versus Q331K:CON by one-way ANOVA with Tukey’s post hoc analysis for multiple comparisons; F(1,4)=3.934, #*P*=0.0171, *n.s.*=not significant by two-sided Student’s *t*-test in **d**.

S**upplementary Fig 2.** Reduction of PP1α phosphorylation in the SOD1 G93A model. **a** p- PP1α immunostaining in spinal MNs of G93A and WT mice at the pre-symptomatic and post-symptomatic stages. Scale bar, 100μm. **b** Analysis of p-PP1α (green) intensity in ChAT (red; MN marker)-positive neurons. WT:Pre=1.00±0.01 (n=3 mice), G93A:Pre=0.86±0.02 (n=4 mice), WT:Post=1.00±0.02 (n=3 mice), G93A:Post=0.68±0.06 (n=3 mice). Values are mean **±**S.E.M. F(1,5)=4.515, ***P*=0.0063 for WT:Pre versus G93A:Pre, ***P*=0.0079 for WT:Post versus G93A:Post by two-sided Student’s *t*-test. **c** Expression of p-PP1α in GFP-positive cortical neurons, following G93A or Q331K overexpression. Arrowhead indicates a GFP-positive neuron. Scale bar, 10μm. **d** The graph shows the quantification of p-PP1α intensity. GFP=100.5 ± 4.38 (n=23 GFP+ neurons), G93A=24.95 ± 4.11 (n=39 GFP+ neurons), Q331K=22.44 ± 4.46 (n=34 GFP+ neurons). Values are mean ±S.E.M. F(2,93)=83.91, ***P<0.001 by one-way ANOVA with Tukey’s post hoc analysis for multiple comparisons.

**Supplementary Fig 3.** Validation of shRNAs against each isoform of PP1 catalytic subunits. Mouse cortical neurons were infected with the lentivirus containing shRNA sequences specific to each catalytic subunit (described on the right). Following three days of infection, endogenous subunit expressions were examined with Western blots. The actin re-blot of the PP1α membrane is shown as a representative.

**Supplementary Fig 4.** Effect of I-2 treatment on the phosphorylation of eIF2a in the SOD1 G93A model at the post-symptomatic stage. p-eIF2a and total eIF2a protein expression levels in the lumbar spinal cord of SOD1 G93A mice were quantified by western blotting.WT=1.00±0.22 (n=3 mice), G93A=1.35±0.29 (n=5 mice). Values are mean **±**S.E.M. F(1,6)=0.8588, n.s.=not significant by two-sided Student’s *t*-test. (N=3 for WT and 5 for G93A biologically independent samples; *n.s*, not significant).
